# Randomized trial of red cell washing for the prevention of transfusion-associated organ injury in cardiac surgery

**DOI:** 10.1093/bja/aex083

**Published:** 2017-05-15

**Authors:** M. J. Woźniak, N. Sullo, S. Qureshi, W. Dott, R. Cardigan, M. Wiltshire, T. Morris, M. Nath, N. Bittar, S. K. Bhudia, T. Kumar, A. H. Goodall, G. J. Murphy

**Affiliations:** 1 Department of Cardiovascular Sciences and NIHR Cardiovascular Biomedical Research Unit, University of Leicester, Glenfield Hospital, Leicester LE3 9QP, UK; 2 National Health Service Blood and Transplant, Cambridge CB2 0PT, UK; 3 Leicester Clinical Trials Unit, Leicester Diabetes Centre, Leicester General Hospital, Leicester LE5 4PW, UK; 4 Blackpool Victoria Hospital NHS Trust, Blackpool, Lancashire FY3 8NR, UK; 5 University Hospitals Coventry and Warwickshire NHS Trust, Clifford Bridge Road, Coventry CV2 2DX, UK

**Keywords:** Cardiovascular surgery, inflammation, transfusion

## Abstract

**Background.** Experimental studies suggest that mechanical cell washing to remove pro-inflammatory components that accumulate in the supernatant of stored donor red blood cells (RBCs) might reduce inflammation and organ injury in transfused patients.

**Methods.** Cardiac surgery patients at increased risk of large-volume RBC transfusion were eligible. Participants were randomized to receive either mechanically washed allogenic RBCs or standard care RBCs. The primary outcome was serum interleukin-8 measured at baseline and at four postsurgery time points. A mechanism substudy evaluated the effects of washing on stored RBCs *in vitro* and on markers of platelet, leucocyte, and endothelial activation in trial subjects.

**Results.** Sixty adult cardiac surgery patients at three UK cardiac centres were enrolled between September 2013 and March 2015. Subjects received a median of 3.5 (interquartile range 2–5.5) RBC units, stored for a mean of 21 (sd 5.2) days, within 48 h of surgery. Mechanical washing reduced concentrations of RBC-derived microvesicles but increased cell-free haemoglobin concentrations in RBC supernatant relative to standard care RBC supernatant. There was no difference between groups with respect to perioperative serum interleukin-8 values [adjusted mean difference 0.239 (95% confidence intervals −0.231, 0.709), *P*=0.318] or concentrations of plasma RBC microvesicles, platelet and leucocyte activation, plasma cell-free haemoglobin, endothelial activation, or biomarkers of heart, lung, or kidney injury.

**Conclusions.** These results do not support a hypothesis that allogenic red blood cell washing has clinical benefits in cardiac surgery.

**Clinical trial registration.** ISRCTN 27076315.

Editor’s key points

- Experimental evidence supports a benefit to red blood cell (RBC) washing to reduce inflammatory factors before transfusion.
- In a randomized trial of washed and standard unwashed RBCs in high-risk cardiac surgery patients, the experimental benefit was not replicated.
- Owing to the limited power of this trial, larger studies are necessary to test the hypothesis that RBC washing is beneficial.

Organ injury associated with red blood cell (RBC) transfusion has been attributed to a ‘storage lesion’; a progressive disruption of erythrocyte homeostasis associated with depletion of high-energy phosphates during storage that results in accumulation of microparticles and other inflammatory substances in the supernatant of RBCs.^1^ Experimental studies implicate platelet and monocyte activation by RBC microparticles, and endothelial dysfunction as a consequence of altered haem metabolism, in transfusion-associated organ injury,^2–5^ and suggest that removal of the storage supernatant by cell washing attenuates inflammatory responses and organ dysfunction.^2^,^6^ In support of these findings, washing of allogeneic RBCs has been shown to attenuate inflammation in children undergoing cardiac surgery.^7^ We tested the hypothesis that allogeneic RBC washing attenuates inflammation and organ failure in adult cardiac surgery patients receiving large-volume transfusions. In a prespecified substudy, we tested the hypothesis that RBC washing attenuates platelet and leucocyte activation by removing inflammatory RBC microparticles. We also assessed whether cell-free haemoglobin (Hgb) release by RBCs after washing results in endothelial activation.

## Methods

The Red Cell Washing for the Attenuation of Organ Injury Following Cardiac Surgery (REDWASH) trial was a multicentre, single-blinded, parallel-group, randomized controlled trial of washing of allogeneic RBCs before transfusion *vs* standard care (no washing). The trial had ethical approval (REC Reference 12/EM/0475) and was registered (ISRCTN 27076315). The trial protocol has been published;^8^ changes to the study design after trial commencement are listed in the online Supplementary material. The main trial was terminated by the funder in March 2015 because of slow recruitment. This report includes the results of a prespecified mechanistic substudy planned for the first 60 patients recruited.^8^ A detailed description of the study methods is available as online Supplementary material.

### Patients

Adults (≥16 yr of age) undergoing cardiac surgery with blood cardioplegia identified as representing a high-risk group for large-volume blood transfusion (LVBT) using a modified risk score^9^ (score ≥25) were eligible for inclusion. Exclusion criteria are listed in Supplementary Table S1.

### Randomization and blinding

Subjects were randomly assigned with concealed allocation using an Internet-based randomization system (Sealed Envelope Ltd, Medicines Healthcare Regulatory Authority (MHRA) recognized facility). Randomization was stratified by study site and type of procedure. Outcome assessors were blinded to allocation.

### Intervention

Eligible subjects who consented to participate were randomly allocated, in a 1:1 ratio, to receive either standard care (unwashed prestorage leucodepleted allogenic red blood cells) or washed red blood cells, between the commencement of surgery and 48 h after surgery. Allogeneic saline–adenine–glucose–mannitol (SAGM) stored RBC units used in the trial were issued by National Health Service Blood and Transplant (NHSBT) as per standard care. The Continuous AutoTransfusion System (CATS™; Fresenius AG, Bad Homburg, Germany) was used on the basis that low ***g*** force centrifugation with this device minimizes RBC trauma.^10^,^11^ For the intervention, RBCs were washed in theatre or at the patient’s bedside with saline using the Quality Mode and immediately administered to the patient. Washed units were not stored for future use. The haematocrit threshold for transfusion was 23. A major protocol violation was defined as receipt of only unwashed blood for subjects randomized to receive washed RBCs, or the receipt of only washed blood in patients randomized to receive standard care.

### Outcomes

The primary outcome was severity of the systemic inflammatory response as indicated by serum interleukin (IL)-8 measured at baseline and at four postsurgery time points. We have previously shown that IL-8 is increased in transfused patients.^8^,^12^ Secondary outcomes are described in Supplementary Table S2. For the mechanism study, serum, platelet-poor plasma, and urine samples were collected at baseline and a serial time points. Microparticles in storage supernatant and platelet-poor plasma were characterized using flow cytometry (Cyan ADP; Beckman Coulter, Brea, CA USA). Cell-free Hgb and plasma total and non-transferrin-bound iron were measured in the supernatant of RBC units and in plasma as described.^13–15^ Platelet activation [platelet activating complex (PAC)-1 (BD Biosciences, Abingdon, Oxford, UK), P selectin/CD62P (Abcam, Cambridge, UK), and PE-coupled CD41 (Affymetrix, Santa Clara, CA, USA)] and leucocyte activation markers (CD64, CD163; Affymetrix) were measured using flow cytometry (Cyan ADP; Beckman Coulter) in whole blood. Platelet activation was also measured indirectly in whole blood using Multiplate aggregometry (Roche Diagnostics International Ltd, Rotkreuz, Switzerland). Serum bilirubin was measured on the Siemens Advia 2400 Chemistry System (Siemens, Frimley, UK). Hepcidin was measured using an enzyme-linked immunosorbent assay (Abbexa, Cambridge, UK). Serum intercellular adhesion molecule (ICAM)-1 was measured using multiplex assays on the MAGPIX (Luminex Corporation, Austin, TX, USA). Reactive oxygen species concentrations, protein carbonyl content, and thiobutiric acid reactive substances (TBARS) were measured with the following commercially available kits: OxiSelect (Cell Biolabs, Inc., San Diego, CA, USA), Parameter TBARS assay (R&D Systems, Abingdon, Oxford, UK), and carbonyl content assay kit (Abcam).

### Statistical analyses

As trial recruitment was terminated early, the comparison of the primary end point (IL-8) is reported here. Secondary outcomes are reported in the online Supplementary material. The mechanism substudy was exploratory; therefore, no sample size calculation was performed. The analysis was performed on an intention-to-treat basis on all randomized subjects who entered the trial (underwent surgery) and had the primary outcome measured at one time point or more (including baseline). Data are presented as the mean (sd) for normally distributed data, median (interquartile range, IQR) for non-normally distributed data, or *n* (%). Means for continuous outcomes for clinical and experimental data (transformed logarithmically if required) were compared using mixed effects models, adjusting for baseline values where available. Treatment estimates were reported as effect sizes with 95% confidence intervals (CIs). A statistical analysis plan was written before database lock (see online Supplementary material). The analysis was performed with SAS version 9.4 (SAS Institute Inc., Cary, NC, USA).

## Results

### Trial cohort

**Fig 1:**
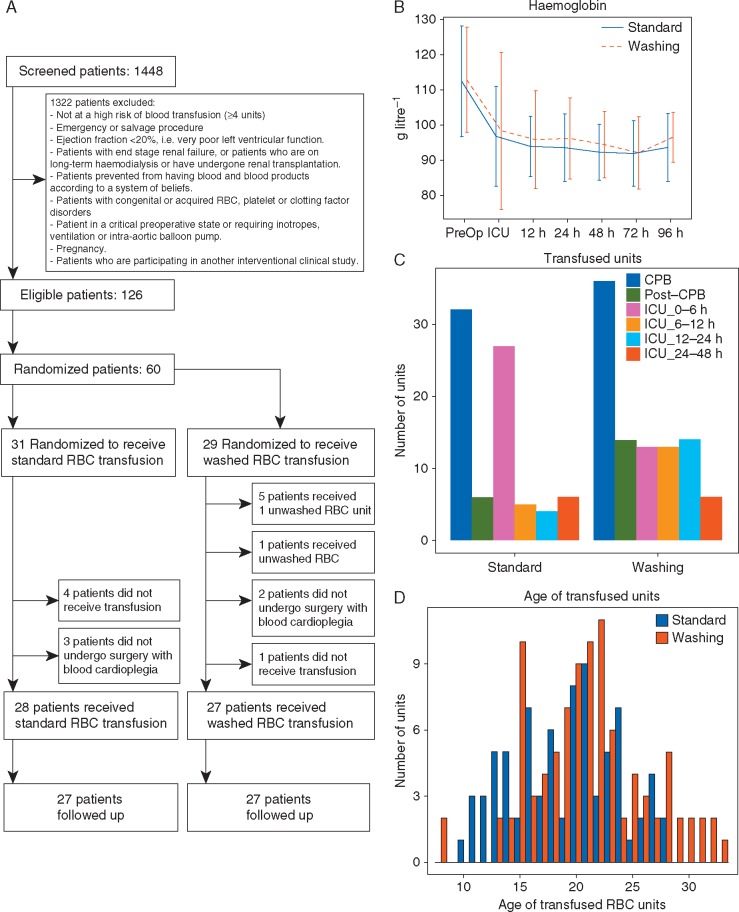
(A) CONSORT diagram of REDWASH trial. (B) Haemoglobin concentrations [mean (sd)] in samples collected from REWASH participants. (C) Number of red blood cell (RBC) units transfused during the trial, during cardiac surgery, and after surgery. (D) Histogram of the transfused RBC unit age distribution.

Sixty subjects were recruited and randomized at three UK centres between May 2013 and March 2015. After withdrawals (Fig. 1A), the analysis population comprised 29 allocated to the standard care arm and 27 to the washing arm. There were four severe protocol deviations, two in the standard care group and two in the washing group. Fifty-four subjects were eligible for follow-up at 3 months. Overall, the mean age of participants was 74 yr [Range 51-90 years], and 28 (50.0%) were female (Supplementary Table S3). Generally, there was good balance between groups; however, by chance there was a lower baseline estimated glomerular filtration rate (eGFR ml .min^-1^. 1.73m^-2^) in the washing group [68 (24) *vs* 90 (30)] in the standard care arm. The types of procedures and the bypass and cross-clamp times were well matched between groups (Supplementary Table S4).

### Transfusion and blood loss

The median number of units transfused was 3 (2–5) in the standard care arm compared with 4 (2–6) in the washing arm (*P*=0.04; Supplementary Table S5). In the washing arm, one subject did not receive any red cells and two received only unwashed RBCs. The distribution of timings (intra- *vs* postoperative) of RBC transfusions was similar between groups (Fig. 1B). The mean storage age of the RBCs was 20 (4.1) and 22 (5.6) days in the standard care and washing arms, respectively (*P*=0.078; Fig. 1C and Supplementary Table S5). There was no difference between groups with respect to blood loss (4 and 12 h; Supplementary Table S5), serial haemoglobin concentrations (Fig. 1D), or exposure to non-RBC blood components (Supplementary Table S5).

### Primary and secondary outcomes

**Fig 2:**
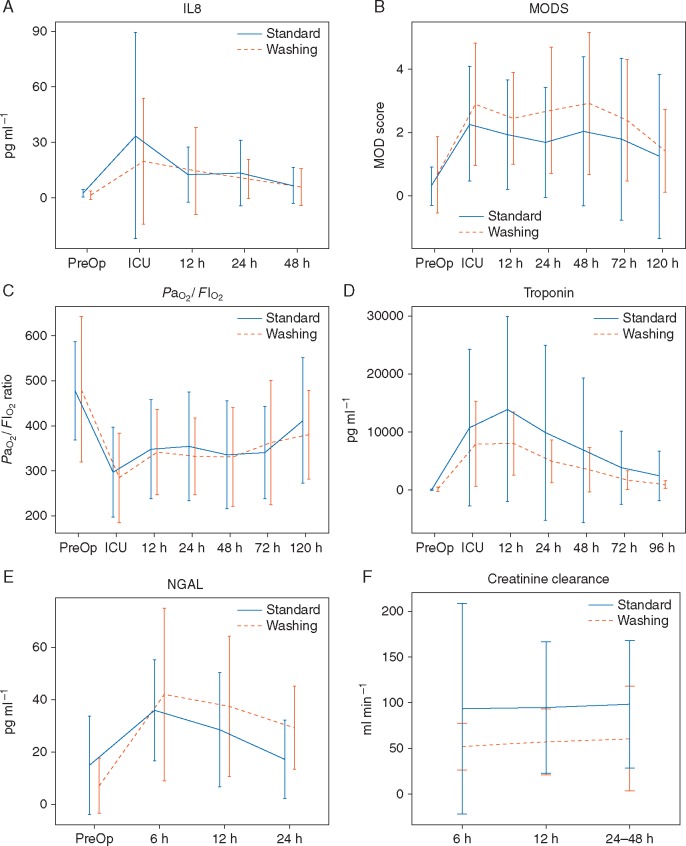
Inflammation and organ injury. (A) Serum interleukin-8 (IL-8). (B) Multiple organ dysfunction scores (MODS). (C) Arterial partial pressure of oxygen/inspired oxygen fraction ($Pa_{O_{2}}/FI_{O_{2}}$) ratio. (D) Serum troponin. (E) Urine neutrophil gelatinase-associated lipocalcin (NGAL). (F) Serum creatinine clearance. Values represent the mean (sd). Findings were not different in prespecified per protocol and safety analyses.

There was no difference between groups for the primary outcome, serum IL-8 [adjusted mean difference, standard *vs* washing, 0.239 (−0.231, 0.709), *P*=0.318; Fig. 2A]. There was no difference between groups with respect to alternative biomarkers of leucocyte and endothelial activation (Supplementary Table S6). There was no difference between groups with respect to MOD scores, worst MOD scores, serial arterial partial pressure of oxygen/inspired oxygen fraction ($Pa_{O_{2}}/FI_{O_{2}}$) ratios, or serial troponin measurements (Fig. 2B–D). Serial creatinine values and urinary neutrophil gelatinase-associated lipocalcin values [logarithmically adjusted mean difference 0.435 (95% CI 0.022, 0.849), *P*=0.039; Fig. 2E] were significantly higher in the washing group, and calculated creatinine clearance was lower (Fig. 2F). However, differences in kidney function and injury were not statistically significant when adjusted for the difference in baseline eGFR (Supplementary Table S7). Clinical outcomes and resource use in the analysis population and adverse events in the safety population are reported for completeness^16^ in Supplementary Tables S8 and S9..

**Fig 3:**
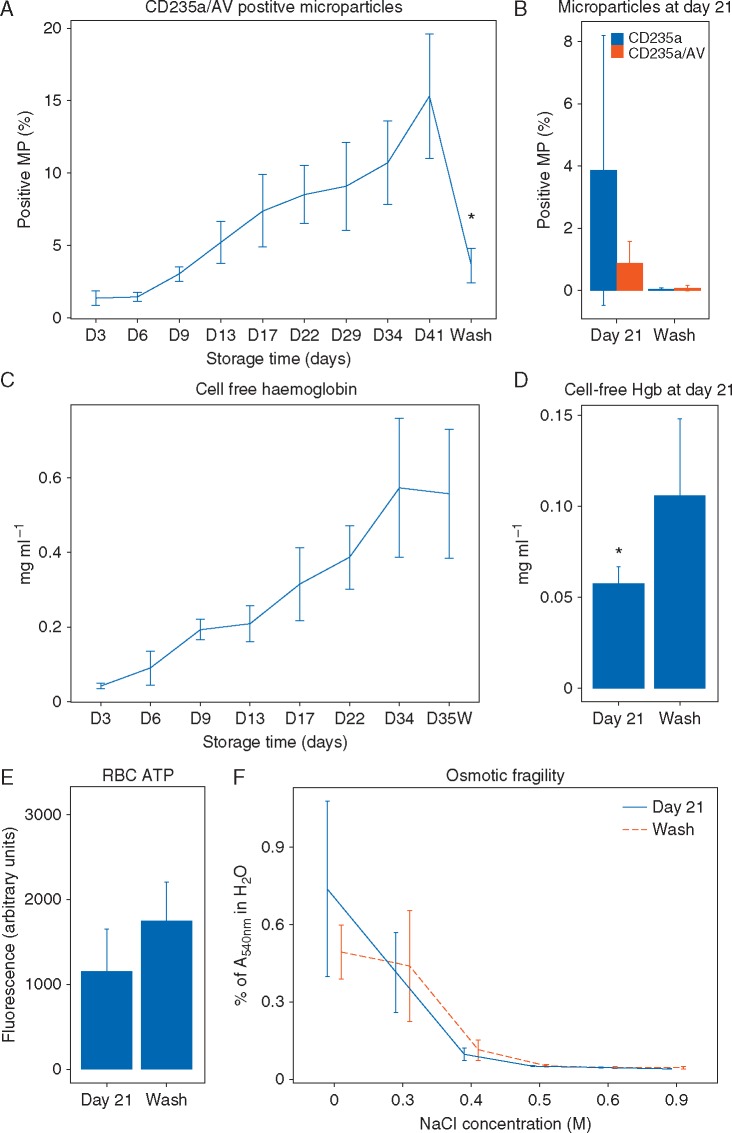
Effects of washing on the red blood cell (RBC) storage lesion *in vitro*. (A)** **Fractional changes in CD235a/annexin V (AV)-positive microparticles during storage and after washing; eight RBC units were analysed. (B) Fractional changes in RBC-derived microparticles in 21-day-stored RBC bags in response to washing (6 units analysed before and 3 units after washing). (C) Cell-free haemoglobin (Hgb) in washed RBC units. (D) Haemoglobin concentrations after washing at day 21. (E) Adenosine triphosphate (ATP) concentrations in 21-day-old RBC bags before and after washing (6 units analysed before and 3 units after washing). (F) Osmotic fragility of 21-day-old RBCs (6 units analysed). Values represent means (sd).

**Fig 4:**
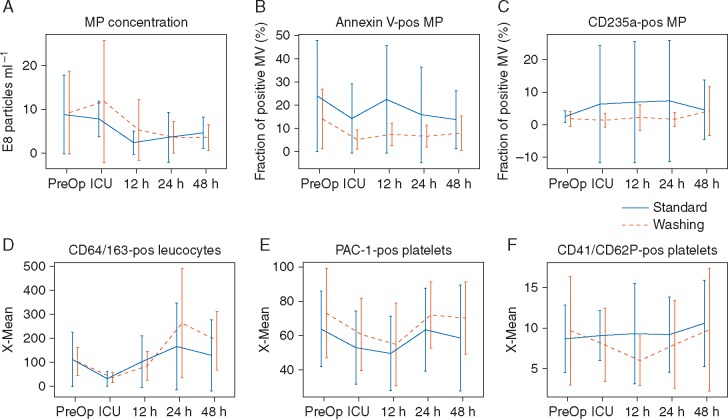
Effects of red blood cell (RBC) washing on microparticle concentrations and platelet and leucocyte activation. (A) Microparticle concentration in collected plasma samples. (B and C) Fractional content of annexin V- (AV) and CD235a-positive microparticles in plasma samples. (D) Activation of monocytes measured by expression of CD64 on monocytes (CD163). (E) Expression of integrin αIIb/β3 (PAC-1) on activated platelets. (F) Expression of CD62P on platelets (CD41). Values represent means (sd).

### Mechanism analyses

#### Effect of washing on stored RBCs

Microparticles positive for CD235a (glycophorin A, an RBC antigen) and annexin V (AV), which labels phosphatidylserine and oxidized lipids, accumulated in the supernatant of RBC units throughout storage (Fig. 3A). Mechanical washing of RBCs significantly reduced concentrations of CD235a/AV microparticle and total microparticle concentrations in units stored for 41 (Fig. 3A) and 21 days (Fig. 3B). Cell-free Hgb concentrations also increased progressively in the supernatant of RBCs during storage (Fig. 3C). Mechanical washing did not reduce concentrations of free Hgb in RBCs stored for 35 days (Fig. 3C) and doubled free Hgb concentrations in RBCs stored for 21 days (Fig. 3D). Washing did not significantly alter RBC adenosine triphosphate concentrations or osmotic fragility (Fig. 3D and E).

#### Effects of RBC washing on microparticles and platelet and leucocyte activation

There was no difference between groups with respect to serial measures of total, AV-positive, or RBC-derived (CD235a positive) microparticles in plasma up to 48 h postsurgery (Fig. 4A–C). There was no difference between groups with respect to concentrations of CD64/CD163-positive (activated) leucocytes, PAC-1, or CD40/CD62P-positive (activated) platelets, as determined by flow cytometry (Fig. 4D–F), or platelet activation as assessed by Multiplate aggregometry (data not shown).

#### Effects of RBC washing on cell-free haemoglobin, oxidative stress, and endothelial activation

**Fig 5:**
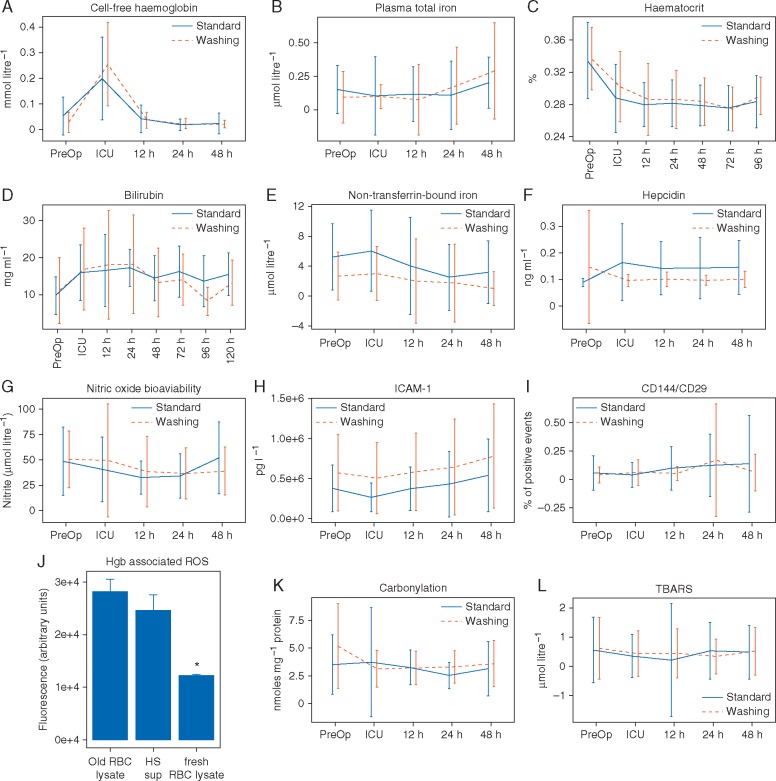
Effects of washing on oxidative stress markers in REDWASH subjects. (A) Plasma cell-free haemoglobin. (B) Plasma total iron concentrations. (C)** **Serial haematocrit. (D) Total bilirubin. (E) Non-transferrin-bound iron. (F) Serum hepcidin. (G) Nitric oxide bioavailability. (H) Serum intercellular adhesion molecule (ICAM)-1. (I)** **Endothelial (CD144) microparticle concentrations in plasma. (J) Oxidative potential of cell-free haemoglobin (Hgb) derived from microparticle-free storage supernatant from stored RBCs (HS Sup) or derived from osmotically lysed stored RBCs or lysed fresh RBCs. ROS, reactive oxygen species. (K) Serum protein carbonylation. (L) Serum thiobarbituric acid reactive substances (TBARS, lipid peroxidation). Values represent means (sd).

Plasma cell-free Hgb concentrations were increased significantly in both washing and standard care groups immediately postsurgery (Fig. 5A). However, there was no difference between groups in plasma free Hgb, iron concentrations, serum haematocrit, serum total bilirubin, non-transferrin-bound iron, hepcidin concentrations, nitric oxide bioavailability, or indicators of endothelial activation [serum ICAM-1 and CD144 (vascular endothelial cadherin)-positive microparticles; Fig. 5B–H]. Specifically, endothelium-derived microparticles did not coexpress integrin β1 (VLA5; Fig. 5I), a putative marker of haem-mediated endothelial activation.

The greater part of plasma free Hgb immediately postsurgery was probably not altered by RBC washing but was most probably attributable to lysis of recipient RBCs by the cardiopulmonary bypass (CPB) circuit. However, free Hgb released after washing of stored RBCs is more reactive than that released by lysed fresh RBCs, as generated by the CPB circuit (Fig. 5J), implying that similar plasma free Hgb concentrations in groups might bely qualitative differences in biological activity. However, we did not detect differences in measures of oxidative stress in serum, a putative mechanism by which free Hgb damages cells, as measured by protein carbonylation or lipid peroxidation (Fig. 5K and L).

## Discussion

Red blood cell-derived (CD235a-positive) microparticles accumulate progressively in RBC units from the start of storage and are effectively removed by mechanical washing. In the REDWASH trial, however, transfusion of washed RBCs did not reduce plasma concentrations of CD235a-positve microparticles or levels of platelet or leucocyte activation in recipients compared with standard RBCs. Cell-free Hgb also increases in the supernatant of RBC units during storage and is increased by washing. However, in the REDWASH trial there was no difference in plasma free Hgb concentrations or markers of haem/iron metabolism, endothelial activation, or oxidative stress between groups.

This is the first randomized trial to evaluate the risks and benefits of RBC washing in adults. We recruited a high-risk population of cardiac surgery patients that should in principle have benefited from safer RBC transfusion. Patients in both groups received significant volumes of RBCs, 1000 ml, within 48 h of surgery and experienced significant morbidity; 86% of patients experienced a composite clinical outcome of death, sepsis, or organ failure. Subjects in the washing group received more RBC units despite similar perioperative levels of blood loss and serial serum haemoglobin concentrations. This is consistent with previous clinical and experimental studies that report loss of 10–25% of red cells during mechanical washing.^6^,^10^ The chief limitations of the study are the limited power of our observations and the early termination of the main trial. For these reasons, we can neither accept nor refute our primary hypotheses. Furthermore, by chance, there was a difference in baseline eGFR between the groups. These limitations notwithstanding, the randomization generated two groups that received large volumes of washed or unwashed RBCs and, after adjustment for baseline eGFR, demonstrated no difference for any measured biomarker of inflammation or organ injury. On balance, our findings argue against important clinical benefits for RBC washing. The REDWASH trial did not evaluate immunomodulation, and therefore, we are unable to comment on the effects of washing on these processes. The results of a similar trial (NCT02094118) evaluating the clinical efficacy of RBC washing will provide greater insights into these results.

Experimental work has documented both potential risks and benefits of RBC washing. We have shown that RBC-derived microparticles activate platelets and leucocytes *in vitro*. Furthermore, we have shown that mechanical RBC washing in a swine model effectively removes microparticles and attenuates platelet and leucocyte sequestration in lungs and the clinical and histological correlates of transfusion-associated lung injury ( MJ Wozniak, GJ Murphy, unpublished observations). Likewise, we have shown that cell-free Hgb activates endothelial cells by a pathway previously described only in human aortic endothelial cells in the presence of minimally modified low-density lipoprotein.^17^ The activation is most probably mediated by lipids oxidized by increased concentrations of free Hgb^18^ that occurs either in the blood bags or after transfusion.^19^ Here, an alternative pathway results in VLA5 (integrin-α5/β1) expression, retention of alternatively spliced CS-1 fibronectin on the surface of endothelial cells, and endothelial cell–monocyte interaction.^20^ In swine, as in humans, RBC washing results in accelerated release of cell-free Hgb, and this results in acute endothelial injury, oxidative stress, and acute kidney injury in experimental subjects (unpublished observations). Studies in human cells have also highlighted potential sublethal damage to RBCs during mechanical washing that could potentially offset any benefits derived from reductions in RBC-derived microparticles.^21^

Our experimental results were not replicated in the REDWASH trial. This could be attributable to differences between species, differences in the time period over which RBCs were transfused, or both, in porcine experiments and trial subjects. Typically, 4 units were transfused during 2 h in swine, whereas a similar volume, on average, was transfused during 2 days in the clinical trial. The porcine experiments were also conducted without CPB. These factors might have diminished the clinical benefits of washing; neither microparticle depletion nor the release of free Hgb observed after washing was reflected by differences in plasma microparticle concentrations or cell-free Hgb in subjects or by differences in inflammation and organ injury. Moreover, the peak mean IL-8 concentration in our porcine experiments after transfusion with older stored RBCs was 10 pg ml^−1^. In the standard care patients in the REDWASH trial, the peak IL-8 concentration was 220 pg ml^−1^. We speculate that the effects of washing were superseded by greater platelet and leucocyte activation, free Hgb release, and endothelial injury attributable to surgery and CPB.^22^ Alternatively, the mixed risks and benefits from RBC washing might have resulted in no overall difference between trial groups. Manual washing techniques that minimize RBC trauma are unlikely to address this limitation because these are time consuming. Indeed, the delay attendant on bedside washing contributed to protocol non-compliance in the present study. An alternative is RBC rejuvenation.^23^ This maximizes the benefits of washing by preventing production of microparticles after washing, minimizes risks by preventing RBC damage and free Hgb release, and attenuate transfusion-mediated organ injury in experimental studies (unpublished observations).

In conclusion, the results of the mechanism substudy of the REDWASH trial did not support our hypothesis that mechanical washing of transfused RBCs would attenuate platelet and leucocyte activation and organ injury in cardiac surgery patients.

## Authors’ contributions

Conceived the trial and wrote the application for funding (with others): G.J.M.

Designed the trial: G.J.M., M.J.W., A.H.G.

Managed the conduct of the trial: W.D., S.K.B., N.B., T.K.

Managed the data during the trial: W.D., T.K., T.M. 

Laboratory analyses: M.J.W., R.C., M.W., N.S.

Statistical analyses: T.M., M.N.

Drafted the report: M.J.W., G.J.M., A.H.G.

All authors reviewed the report for important intellectual content and approved the final version. 

All authors, external and internal, had full access to all of the data (including statistical reports and tables) and can take responsibility for the integrity of the data and the accuracy of the data analysis.

## Supplementary material

Supplementary material is available at *British Journal of Anaesthesia* online.

## Supplementary Material

Supplementary Data
