# Supplementary material for: Randomized trial of red cell washing for the prevention of transfusion-associated organ injury in cardiac surgery
Source: Br J Anaesth. 2017 May 15;118(5):689–98. doi: 10.1093/bja/aex083 (PMC5430295; doi:10.1093/bja/aex083)
Supplement: Supplementary Data [file aex083_Supp.docx]

# eSupplement: Red cell washing for the prevention of transfusion associated organ injury in cardiac surgery: The REDWASH Trial

Woźniak MJ, Sullo N, Qureshi S, Dott W, Cardigan R, Wiltshire M, Morris T, Nath M, Bittar N, Bhudia S, Kumar T, Goodall AH and Murphy GJ

# Supplemental Materials and Methods

**Red Cell Analyses:** For the in vitro washing studies eight randomly (blood groups 4x O Rh+, 4x AB Rh+) selected routinely processed, packed human red blood cell units were obtained from NHS Blood and Transplant. Red cell washing procedures were performed on 21-day, and 35-day old RBC units using the quality mode of the continuous autotransfusion system (CATS^TM^, Fresenius AG, Bad Homburg, Germany) on the morning of the experiment. The washing procedure lasted approximately 20 min and used 3000 – 5000mL of 0.9% sodium chloride wash solution for up to 4 units of red cells (Baxter BV, Utrecht, the Netherlands).The device was setup and used according to the manufacturers recommended protocol.

**Biochemical markers analysis** Interleukin (IL)-6, IL-8, Tumour Necrosis Factor (TNF)α, Macrophage Inflammatory Protein (MIP)-1, Monocyte Chemotactic Protein (MCP)-1 and Intercellular Adhesion Molecule (ICAM)-1 in human serum samples were assessed using Affymetrix (Santa Clara, CA) ProcartaPlex Immunoassay kits according to manufacturer’s recommendations using the Magpix system (Luminex, Austin, TX, USA). Urine concentrations of neutrophil gelatinase-associated lipocalin (NGAL) were measured using the human NGAL ELISA kit from EKF-diagnostics (Barleben, Germany). Hepcidin levels were measured in human serum samples using a commercial ELISA kit from Abbexa (Cambridge, UK), on the DS2® 2-Plate ELISA Processing System (Dynex Technologies, Chantilly, VA). ICAM-1 was quantified quantified in serum samples by multiplex assays (Affymetrix, Santa Clara, CA) on the Magpix device (Luminex Corporation, Austin, TX). Nitric oxide (NO) bioavailability in human plasma was measured using a colorimetric R&D Systems (Abingdon, UK) kit and EnSpire® Multimode Plate Reader (PerkinElmer, Waltham, MA). Reactive oxygen species levels, protein carbonyl content and thiobutiric acid reactive substances (TBARS) were measured with a commercially available kits: OxiSelect (Cell Biolabs, Inc., San Diego, CA), Parameter TBARS assay (R&D Systems) and carbonyl content assay kit (Abcam, Cambridge, UK)

**Biochemical and biophysical analysis of RBC bags** Adenosine triphosphate (ATP), 2, 3 DPG, and reactive oxygen species levels were measured with a commercially available kits (ATPlite, PerkinElmer, 2, 3 DPG, Roche, and OxiSelect, CellBiolabs, San Diego, CA; respectively) using NovoStar reader (BMG LabTech, Offenburg, Germany). For these assays, RBC samples were deproteinised with perchloric acid as described previously ^1^. The haemolysis index of donor red cells was defined as (Plasma haemoglobin x Hematocrit)/Donor Unit haemoglobin. For osmotic fragility 0.1 mL of packed RBCs was added into tubes containing 10 mL of increasing concentration of buffered salt solution (pH 7.4; NaCl (%) 0, 0.3, 0.4, 0.5, 0.6, 0.9). The tubes were gently mixed and incubated at room temperature for 20 min. The samples were spun at 1500 × g for 10 min and the supernatant absorbances were measured at 540 nm using a plate reader (Enspire, Perkin Elmer). Haemolysis in each tube was expressed as percentage of the absorbance in distilled water.

**Leukocyte and platelets analysis** in human samples was done immediately after collection: For activated leucocytes 100 μL of whole blood was labelled with FITC-coupled CD64 and PE-coupled CD163 (Affymetrix, Santa Clara, CA) antibodies at 1:20 dilution for 25 min at room temperature (RT). Red cells were lysed with FACS Lysing Solution (BD Biosciences, Oxford, UK) for 10 min at RT, leukocytes were spun at 3000 rpm in a bench top centrifuge and washed with 1 mL PBS. The pellet was resuspended in 300 μL PBS and analysed using Cyan ADP flow cytometer driven by Summit V4.3.02 Build 2451 (Beckman Coulter, Brea, CA). For platelets analysis, 5 μL of whole blood was labelled with FITC-coupled Platelet activating Complex (PAC)-1 (BD Biosciences), E-Selectin/CD62E (Abcam, Cambridge, UK) and PE-coupled CD41 (Affymetrix) antibodies at 1:20 dilution at RT for 25 min in total volume of 100 μL. Samples were analysed using CyAn ADP flow cytometer.

For Multiplate analyses (Roche Diagnostics International Ltd, Rotkreuz, Switzerland) whole blood was collected into 3.0-mL tubes containing hirudin, a direct thrombin inhibitor anticoagulant. Then 300 μL blood was added to 300 μL of 37°C pre-heated saline solution and platelet aggregation was analysed after activation with commercially available adenosine diphosphate (ADP, ADP test, 6.5-μM final concentration). Increasing electric impedance was electronically measured for 6 minutes and expressed as the area under the aggregation curve plotted over time (AUC U) by an integrated software program. Blood collection tubes, test cells, reagents, and analysing software were all provided by the manufacturer as a part of the standard supply to our hospital.

**Microparticle (MP) analysis** was done in citrated plasma samples spun twice at 1500 xg and red cell bags supernatant spun at 1850 xg after 1:1 dilution with PBS. Concentration and size distribution were estimated using NanoSight NS500, nano-particle tracking device (Malvern Instruments, Malvern, UK). Derivation of MP and phosphatidylserine (PS) exposure were determined with FITC-coupled CD235a (glycophorin A, a red cell antigen), CD14 (a macrophage/ monocyte marker) and CD284 (Toll-like receptor 4) antibodies, and annexin V (PE-coupled, a phospahtadylserine/ oxidised lipid marker) (Affymetrix): 20 – 50 μL samples were labelled with antibodies at 1:20 dilution in annexin V binding buffer for 25 min at RT in total volume 100 μL. The samples were analysed by flow cytometry (CyAn ADP). To separate larger MP, RBC supernatant was further spun at 28,000 xg for 35 min. The pellet was re-suspended in PBS and stored at -80C, as was the remaining high speed supernatant (HS sup), for later analyses.

**Free haemoglobin and iron metabolism** Cell-free haemoglobin was estimated in HS sup and plasma as described ^2^. Briefly, the samples were spun at 1,800 xg for 15 min and the resulting supernatant was further spun for 5 min at 20,000 xg. The supernatant was diluted 5x with PBS and absorbance measured at 415nm, 450nm and 700nm using Multiplate reader Enspire (PerkinElmer). Haemoglobin concentration was calculated as follows: Hgb = 1.58xA_415_ - 0.95xA_450_ - 2.91xA_700_. Free iron was measured in plasma samples as previously described ^3^: 73.5µl of plasma was incubated with 1.5µl of 50mM ferrous iron chelator BPS (bathophenanthroline disulfonate) for 15 min. Absorbance was measured at 535 nm and compared against standard curve prepared with ferrous ammonium sulphate. Non-transferrin-bound iron was chelated with 80 mM nitrilotriacetic acid (NTA) for 30 min at RT and filtered through Amicon Ultra filters (Merck Millipore, Billerica, MA) , MW 30 000 cutoff according to manufacturer’s recommendations. The filtrate was diluted 1:1 with 5 mM MOPS buffer (pH 7.4) and incubated with 12 mM thioglycolic acid and 6 mM BPS for 30 min at RT. Absorbance was measured at 537 nm ^4^.

**Statistical Analyses**

The REDWASH trial was terminated by the funder because of slow recruitment. This report includes the results of a pre-specified mechanistic sub-study planned for the first 60 patients recruited in the trial [Murphy 2012]. The mechanism sub-study was exploratory therefore no sample size calculation was performed. The analysis was performed on an intention-to-treat basis on all randomised patients who entered the trial (underwent surgery) and had the primary outcome measured at least one time point (including baseline). Means for continuous outcomes for clinical and experimental data (transformed logarithmically if required) were compared using mixed effects models, adjusting for baseline values where available. Findings were reported as effect sizes with 95% confidence intervals. A Statistical Analysis Plan giving was written prior to database lock [see appendix]. The analysis was performed with SAS version 9.4 (SAS Institute Inc., Cary, NC, USA). For some REDWASH variables we evaluated the Box-Cox power transformation of each physiological and immunological variable, and if required, identified the appropriate transformation function to account for the increased variability of the variable with the corresponding mean. Each of the transformed (or un-transformed) physiological and immunological variable was analysed using a linear mixed model incorporating available baseline variables and group as fixed effects. The model included a random intercept for each patient, and if necessary, a random time-specific slope for each patient. Additionally, we explored different variance structures to deal with the residual heterogeneity in the data. For the binary data on COPD, ARDS, Non-ALI, ALI-Non-ARDS, we fitted a logistic mixed model with a logit link function assuming the Bernoulli distribution of the individual variable. The model included group and time as fixed effects and patient as a random effect. We fitted cumulative link mixed model on the ordinal data of Berlin Score and AKI; the model incorporated group as a fixed effect and patient as a random effect. All statistical analyses were carried out in the R software environment (R Core Team, 2015) with appropriate packages (nlme, lme4, ordinal).

# Acknowledgements

List of investigators and research team members for each centre: Glenfield Hospital Leicester PI: Prof Gavin Murphy, Trial Coordinator: Dr William Dott, previously Ms Pia Nielsen, Trial Manager: Mrs Tracy Kumar, Science Lead: Dr Marcin Woźniak, Dr Nikol Sullo, Research Nurse: Mrs Latisha Joel-Davies

University Coventry and Warwick NHS Foundation Trust PI: Mr Sunil Bhudia

Blackpool Royal Victoria Hospital PI: Mr Nidal Bittar, Research Nurses:

The authors would like to thank members of the Cardiac Surgery Programme Steering Committee who acted as the Trial Steering Committee for the REDWASH trial: Professor Alison Goodall, Professor Nigel Brunskill, Dr Karl Herbert, Mr Sunil Bhudia, Mr Alan Philipps and Mr Anthony Locke.

# eReferences

1. Wilsher C, Garwood M, Sutherland J, Turner C and Cardigan R. The effect of storing whole blood at 22 degrees C for up to 24 hours with and without rapid cooling on the quality of red cell concentrates and fresh-frozen plasma. *Transfusion*. 2008;48:2338-47.

2. Fairbanks VF, Ziesmer SC and O'Brien PC. Methods for measuring plasma hemoglobin in micromolar concentration compared. Clin Chem. 1992;38:132-40.

3. Nilsson UA, Bassen M, Savman K and Kjellmer I. A simple and rapid method for the determination of "free" iron in biological fluids. *Free radical research*. 2002;36:677-84.

4. Gosriwatana I, Loreal O, Lu S, Brissot P, Porter J and Hider RC. Quantification of non-transferrin-bound iron in the presence of unsaturated transferrin. *Analytical biochemistry*. 1999;273:212-20

# eTable 1. REDWASH Trial Exclusion criteria

| Participant could not enter study if ANY of the following applied: |
| --- |
| • Emergency or salvage procedure. |
| • Ejection fraction <20%, i.e. very poor left ventricular function. |
| • Patients with end stage renal failure defined as an estimated Glomerular Filtration rate (eGFR) <15ml min^-1^ 1.72m^-2^ calculated from the Modification of Diet in Renal Disease equation [32], or patients on long-term dialysis or who have undergone renal transplantation. |
| • Patients prevented from having blood and blood products according to a system of beliefs (e.g. Jehovah’s Witnesses). |
| • Patients with congenital or acquired RBC, platelet or clotting factor disorders, (excluding those receiving antiplatelet therapy, warfarin or other systemic oral anticoagulants). |
| • Patient in a critical preoperative state (Kidney Disease: Improving Global Outcomes (KDIGO) Stage 3 AKI [33] or requiring ionotropes, ventilation or intra-aortic balloon pump) preoperatively. |
| • Pregnancy |
| • Patients participating in another interventional clinical study. |

# eTable 2. REDWASH Trial Clinical and Secondary Outcomes [from Murphy 2016]

| **Outcome** | **Definition/ Method of Verification** |
| --- | --- |
| **Primary outcome** | **Serum cytokine IL-8 at return to ITU, 6-12 hours, 24 hours, and 48 hours.** |
| Inflammatory Organ Injury, Sepsis or Death | - Sepsis was defined as Antibiotic treatment for suspected infection, **and** the presence of SIRS within 24 hours prior to start of antibiotic treatment where SIRS is defined as It was defined as ≥ 2 of the following conditions: temperature > 38 ^o^C or < 36 ^o^C; heart rate > 90 beats per min; respiratory rate > 20 breaths per min or PaCO2 < 32 mmHg; white blood cell count > 12,000 per mm^3^ or < 4,000 per mm^3^, **or** antibiotic treatment for wound infection. - Acute Kidney Injury, defined as KDIGO [33] stage 1, 2 or 3. - Acute lung injury, defined as PaO2/FiO2 ratio <300mmHg or a requirement for respiratory support; invasive ventilation>48hours, non-invasive ventilation>4 hours, re-intubation, tracheostomy, or ARDS. - Low cardiac output, defined as new intra-or postoperative intra-aortic balloon pump insertion or a cardiac index of <2.2 L · min−1 · m−2 refractory to appropriate intravascular volume expansion after correction or attempted correction of any dysrhythmias, or the administration of ionotropes including Dobutamine, Enoximone, Milrinone, Levosimendan and Adrenaline. - Death - Differences in Multiple Organ Dysfunction Score [35] at days 1,2, 3 and 5. |
| Bleeding and Transfusion | - Blood loss at 6 hours postoperatively. - The number of units of RBC and other blood components transfused during the operative period and post-operative hospital stay was recorded |
| Transfusion Reactions | - Febrile Transfusion Reactions - Non-haemolytic transfusion reactions. - Haemolytic Transfusion reactions. |
| Other clinical outcomes | - Stroke; diagnosed by brain imaging (CT or MRI), in association with new onset focal or generalised neurological deficit (defined as deficit in motor, sensory or co-ordination functions) - ST elevation myocardial infarction accompanied by troponin I > 5000 pg ml^-1^ |
| Hospital stay and cumulative resource use | ITU, HDU and hospital length of stay were determined by the assessment of care level. |
| Compliance with the washing protocol | Data was collected for all patients during surgery to characterise compliance with the randomly assigned washing protocol. |
| Mechanism Sub-Study (first 60 patients) | - Neutrophil gelatinase associated lipocalin (NGAL) at baseline and at 6, 12 and 24 hours - Serum Troponin I at baseline and at 24 and 48 hours. - Age of each unit of RBC transfused. - Serum levels of IL-6, MCP-1, MIP-1 and TNF-α at the same time points as for the primary endpoint. - Platelet and monocyte activation as determined by flow cytometry - Endothelial injury as determined by quantification of endothelial derived microparticles by flow cytometry. |

# eTable 3: REDWASH trial participant demographics and past history

|  | | | | |  | **Randomised patients** | | | | | |
| --- | --- | --- | --- | --- | --- | --- | --- | --- | --- | --- | --- |
| **Characteristic** | | | | |  | **Standard care (N=29)** | | **Red cell washing (N=27)** | | **Overall (N=56)** | |
|  | | | | |  | **N** | **%** | **N** | **%** | **n** | **%** |
| DEMOGRAPHY | | | | | | | | | | | |
| Gender [Female] | | | | | | 15 | 51.7% | 13 | 48.1% | 28 | 50.0% |
| Age [Years] (Mean, SD) | | | | | | 74.6 | (8.9) | 74.1 | (9.2) | 74.4 | (9.0) |
| BMI (Mean, SD) | | | | | | 28.9 | (4.8) | 29.2 | (6.1) | 29.1 | (5.4) |
| NYHA class | | I | | | | 5 | 17.2% | 4 | 14.8% | 9 | 16.1% |
|  | | II | | | | 17 | 58.6% | 11 | 40.7% | 28 | 50.0% |
|  | | III | | | | 6 | 20.7% | 12 | 44.4% | 18 | 32.1% |
|  | | IV | | | | 1 | 3.4% | 0 | 0.0% | 1 | 1.8% |
| CSS class | | Asymptomatic | | | | 14 | 48.3% | 13 | 48.1% | 27 | 48.2% |
|  | | I | | | | 7 | 24.1% | 8 | 29.6% | 15 | 26.8% |
|  | | II | | | | 6 | 20.7% | 2 | 7.4% | 8 | 14.3% |
|  | | III | | | | 2 | 6.9% | 3 | 11.1% | 5 | 8.9% |
|  | | IV | | | | 0 | 0.0% | 1 | 3.7% | 1 | 1.8% |
|  | | | | | | | | | | | |
| LV function | Good (>50%) | | | | |  |  |  |  |  |  |
|  | Moderate (30-50%) | | | | |  |  |  |  |  |  |
|  | | | | | | | | | | | |
| Haemoglobin [g dl^-1^] Mean (SD) | | | | | | 11.1 | (1.5) | 11.2 | (1.4) | 11.2 | (1.4) |
| Haematocrit [%] Mean (SD) | | | | | | 33.0 | (4.6) | 33.5 | (3.6) | 33.2 | (4.1) |
| Platelets [10^9^ l^-1^] Mean (SD) | | | | | | 263.7 | (123.6) | 245.5 | (67.6) | 254.6 | (99.1) |
| Creatinine [mmol l^-1^] Mean (SD) | | | | | | 6.8 | (3.9) | 6.8 | (4.3) | 6.8 | (4.1) |
| Estimated Glomerular Filtration rate [ml min^-1^ 1.^73m-2^] Mean (SD) | | | | | | 88.5 | (29.7) | 68.5 | (24.5) | 78.9 | (28.9) |
|  | | | | | | | | | | | |
| Diabetic | | | No | | | 14 | 48.3% | 17 | 63.0% | 31 | 55.4% |
|  | | | Diet | | | 3 | 10.3% | 2 | 7.4% | 5 | 8.9% |
|  | | | Oral | | | 9 | 31.0% | 4 | 14.8% | 13 | 23.2% |
|  | | | Insulin | | | 3 | 10.3% | 4 | 14.8% | 7 | 12.5% |
| Pacemaker | | | No | | | 26 | 89.7% | 25 | 92.6% | 51 | 91.1% |
|  | | | Temporary | | | 0 | 0.0% | 0 | 0.0% | 0 | 0.0% |
|  | | | Permanent | | | 2 | 6.9% | 2 | 7.4% | 4 | 7.1% |
| CVA or TIA | | |  | | | 3 | 10.3% | 4 | 14.8% | 7 | 12.5% |
| Smoking status | | | No | | | 13 | 44.8% | 4 | 14.8% | 17 | 30.4% |
|  | | | Ex (>1 month) | | | 13 | 44.8% | 20 | 74.1% | 33 | 58.9% |
|  | | | Yes | | | 1 | 3.4% | 0 | 0.0% | 1 | 1.8% |
| Previous cardiac surgery | | | | | | 3 | 10.3% | 5 | 18.5% | 8 | 14.3% |
| Myocardial infarction | | | | | | 4 | 13.8% | 4 | 14.8% | 8 | 14.3% |
| Operative priority | | | | Elective | | 15 | 51.7% | 13 | 48.1% | 28 | 50% |
|  | | | | Urgent | | 14 | 48.3% | 14 | 51.9% | 28 | 50% |
| Logistic EuroScore (Median, IQR) | | | | | | 9.6 | (7.1, 19.1) | 10.7 | (6.0, 23.5) | 10.0 | (6.6, 19.3) |
| LVBT Score (Median, IQR) | | | | | | 33.1 | (30.0, 39.2) | 34.6 | (31.2, 41) | 34.0 | (28.8, 40.0 |
| **MEDICATIONS** | | | | | | | | | | | |
| Heparin | | | | | | 0 | 0.0% | 0 | 0.0% | 0 | 0.0% |
| Clexane within 12 hours preoperatively | | | | | | 1 | 3.7% | 0 | 0.0% | 1 | 1.8% |
| Aspirin within 5 days preoperatively | | | | | | 15 | 51.7% | 16 | 59.3% | 31 | 55.4% |
| Clopidogrel within 5 days preoperatively | | | | | | 3 | 10.3% | 3 | 11.1% | 6 | 10.7% |

# eTable 4: Operative characteristics

|  | | | | **Randomised patients** | | | | | |
| --- | --- | --- | --- | --- | --- | --- | --- | --- | --- |
| **Characteristic** | | | | **Standard Care (N=29)** | | **Red cell washing (N=27)** | | **Overall (N= 56)** | |
|  | | | | **n** | **%** | **N** | **%** | **N** | **%** |
| Type of surgery | CABG | | | 4 | 13.8% | 6 | 22.2% | 10 | 17.9% |
|  | Valve | | | 10 | 34.5% | 9 | 33.3% | 19 | 33.9% |
|  | CABG and valve | | | 10 | 34.5% | 9 | 33.3% | 19 | 33.9% |
|  | Other | | | 5 | 17.2% | 3 | 11.1% | 8 | 14.3% |
| **Conduct of Cardiopulmonary Bypass** | | | | **Mean** | **(SD)** | **Mean** | **(SD)** | **Mean** | **(SD)** |
| Bypass duration (hours) Mean (SD) | | | | 2.1 | (0.9) | 1.9 | (0.8) | 2.0 | (0.9) |
| Cross clamp duration (hours)  Mean (SD) | | | | 1.3 | (0.6) | 1.3 | (0.7) | 1.3 | (0.6) |
| Myocardial protection N (%) | | Blood | | 29 | 100% | 27 | 100% | 56 | 100% |
|  | | Crystalloid | | 0 |  | 0 |  | 0 |  |
| Baseline Values/ CPB Start | | |  |  |  |  |  |  |  |
| MABP | | |  | 60.1 | (10.8) | 57.6 | (13.3) | 58.9 | (12.0) |
| Hct | | |  | 24.1 | (5.2) | 23.4 | (4.7) | 23.7 | (4.9) |
| Lactate | | |  | 1.5 | (1.3) | 1.5 | (2.0) | 1.5 | (1.6) |
| **20 min CPB time** | | |  |  |  |  |  |  |  |
| MABP | | |  | 58.3 | (9.3) | 60.6 | (6.5) | 59.4 | (8.1) |
| Hct | | |  | 23.8 | (2.7) | 22.6 | (3.8) | 23.2 | (3.3) |
| Lactate | | |  | 1.8 | (1.2) | 1.7 | (2.1) | 1.7 | (1.7) |
| **40 min CPB time** | | |  |  |  |  |  |  |  |
| MABP | | |  | 60.8 | (8.8) | 62.7 | (8.4) | 61.7 | (8.6) |
| Hct | | |  | 25.2 | (2.9) | 24.1 | (3.4) | 24.7 | (3.1) |
| Lactate | | |  | 1.9 | (1.4) | 2.0 | (2.7) | 2.0 | (2.1) |
| **Pre-CPB Rewarming** | | |  |  |  |  |  |  |  |
| MABP | | |  | 61.6 | (10.8) | 62.4 | (9.0) | 62.0 | (10.0) |
| Hct | | |  | 25.4 | (2.9) | 24.2 | (3.2) | 24.8 | (3.1) |
| Lactate | | |  | 2.0 | (1.5) | 2.1 | (2.9) | 2.1 | (2.2) |
| **Pre-CPB Weaning** | | |  |  |  |  |  |  |  |
| MABP | | |  | 60.9 | (9.5) | 64.1 | (11.5) | 62.5 | (10.6) |
| Hct | | |  | 26.4 | (2.2) | 25.3 | (2.7) | 25.8 | (2.5) |
| Lactate | | |  | 2.2 | (1.5) | 2.5 | (2.8) | 2.3 | (2.3) |
| **Values on Return to ITU** | | |  |  |  |  |  |  |  |
| MABP | | |  | 76.4 | (11.9) | 71.1 | (16.2) | 73.9 | (14.2) |
| Hct | | |  | 27.7 | (4.3) | 28.8 | (3.9) | 28.2 | (4.2) |
| Lactate | | |  | 2.6 | (1.7) | 3.7 | (3.7) | 3.1 | (2.9) |
| Temperature | | |  | 35.8 | (0.9) | 36.1 | (0.7) | 35.9 | (0.8) |
| PaO2 | | |  | 17.3 | (6.1) | 16.4 | (5.5) | 16.9 | (5.8) |
| PaCO2 | | |  | 5.4 | (0.7) | 5.7 | (0.7) | 5.5 | (0.7) |

# eTable 5. Red cell transfusion and blood loss

|  | **Randomised patients** | | | | | |  |  |
| --- | --- | --- | --- | --- | --- | --- | --- | --- |
|  | **Standard care (N=29)** | | **Red cell washing (N=27)** | | **Overall (N=56)** | |  |  |
|  | **N** | **%** | **n** | **%** | **n** | **%** | **Effect (95% CI)** | **p-value** |
| **Units Transfused** |  |  |  |  |  |  |  |  |
| 0 | 3 | 10.3% | 1 | 3.7% | 4 | 7.1% |  |  |
| 1 | 3 | 10.3% | 1 | 3.7% | 4 | 7.1% |  |  |
| 2 | 5 | 17.2% | 5 | 18.5% | 10 | 17.9% |  |  |
| 3 | 6 | 20.7% | 4 | 14.8% | 10 | 17.9% |  |  |
| 4 | 4 | 13.8% | 4 | 14.8% | 8 | 14.3% |  |  |
| >4 | 8 | 27.6% | 12 | 44.4% | 20 | 35.7% |  |  |
| Total units transfused | 98 | - | 123 | - | 221 | - |  |  |
| **by setting** |  |  |  |  |  |  |  |  |
| In Theatre - median (IQR) | 1 | (0-2) | 2 | (0-3) | 2 | (0-2) |  |  |
| In ICU - median (IQR) | 2 | (1-3) | 3 | (1-4) | 2 | (1-4) |  |  |
| Total Units - median (IQR) | 3 | (2-5) | 4 | (2-6) | 3.5 | (2-5.5) | 1.32* (1.01, 1.73) | 0.040 |
| Storage age (mean (SD)) | 19.7 | (4.08) | 21.7 | (5.6) | 21.0 | (5.2) |  | 0.078 |
| **Blood Loss [ml]** |  |  |  |  |  |  |  |  |
| 4 hours – mean (SD) | 228.3 | 144.5 | 251.9 | 174.5 | 239.7 | 158.6 | 28.08†  (-109.02, 52.86) | 0.489 |
| 12 hours – mean (SD) | 470.1 | 258.2 | 497.1 | 253.3 | 483.1 | 253.9 | 27.71†  (-154.67, 99.26) | 0.663 |
| **Non red cell components**  **–median (IQR)** | 0.5 | (0-4) | 2 | (0-5) | 1 | (0-5) | 1.01* (0.71, 1.44) | 0.939 |
| **Transfusion reactions** | 0 |  | 0 |  | 0 |  |  |  |

*Count ratio from a Poisson generalized linear model

†Adjusted mean

# eTable 6. Biomarkers of inflammation

|  | **Randomised patients** | | | |  |  |
| --- | --- | --- | --- | --- | --- | --- |
|  | **Standard care** | | **Red cell washing** | |  |  |
|  | **Mean** | **SD** | **Mean** | **SD** | **Effect (95% CI)** | **p-value** |
| **Log(Serum IL-8)** | **N=29** |  | **N=26** |  |  |  |
| Baseline | 0.659 | 1.083 | -0.105 | 1.243 |  |  |
| Return to ICU | 2.409 | 1.382 | 1.730 | 1.543 | -0.193  (-0.894, 0.509) | 0.591 |
| 6-12 hours | 1.942 | 1.289 | 1.785 | 1.205 | 0.367 (-0.202, 0.936) | 0.207 |
| 24 hours | 1.906 | 1.343 | 1.772 | 1.163 | 0.361 (-0.224, 0.945) | 0.226 |
| 48 hours | 1.228 | 1.204 | 1.072 | 1.147 | 0.393 (-0.166, 0.952) | 0.166 |
| **Full analysis population with imputed values***  Overall effect |  |  |  |  | 0.239 (-0.231, 0.709) | 0.318 |
| **Full analysis population: complete cases**  Overall effect |  |  |  |  | 0.262 (-0.265, 0.788) | 0.322 |
| **Per protocol population**†  Overall effect | N=26 |  | N=23 |  | 0.182 (-0.318, 0.683) | 0.475 |
| **Log(TNFα)** | N=23 |  | N=22 |  |  |  |
| Baseline | -1.033 | 1.718 | -1.324 | 1.720 |  |  |
| Return to ICU | -0.058 | 1.734 | -0.915 | 1.773 | -0.679 (-1.545, 0.187) | 0.121 |
| 6-12 hours | -0.760 | 1.581 | -1.281 | 1.650 | -0.311 (-1.049, 0.427) | 0.399 |
| 24 hours | -0.810 | 1.912 | -0.932 | 1.962 | 0.089 (-0.637, 0.814) | 0.806 |
| 48 hours | -0.607 | 1.789 | -1.354 | 1.654 | -0.558 (-1.467, 0.351) | 0.228 |
| Overall effect |  |  |  |  | -0.362 (-1.022, 0.299) | 0.283 |
| **Log(Serum IL 6)** | **N=23** |  | **N=22** |  |  |  |
| Baseline | 2.309 | 1.871 | 1.940 | 1.860 |  |  |
| Return to ICU | 5.401 | 1.729 | 5.549 | 1.603 | 0.323 (-0.512, 1.158) | 0.448 |
| 6-12 hours | 5.266 | 1.250 | 5.513 | 1.223 | 0.400 (-0.197, 0.998) | 0.189 |
| 24 hours | 5.035 | 1.036 | 5.272 | 0.995 | 0.355 (-0.148, 0.857) | 0.167 |
| 48 hours | 4.702 | 0.950 | 4.681 | 1.142 | 0.108 (-0.389, 0.606) | 0.670 |
| Overall effect |  |  |  |  | 0.310 (-0.173, 0.792) | 0.209 |
| **Log(MCP-1)** | N=23 |  | N=22 |  |  |  |
| Baseline | 4.309 | 0.834 | 4.061 | 1.507 |  |  |
| Return to ICU | 5.772 | 1.038 | 4.476 | 2.845 | -0.933 (-1.977, 0.110) | 0.078 |
| 6-12 hours | 4.955 | 0.978 | 4.637 | 1.804 | -0.339 (-1.239, 0.561) | 0.451 |
| 24 hours | 4.679 | 1.100 | 4.060 | 2.132 | -0.640 (-1.685, 0.404) | 0.222 |
| 48 hours | 4.380 | 0.937 | 3.884 | 2.403 | -0.458 (-1.567, 0.650) | 0.407 |
| Overall effect |  |  |  |  | -0.638 (-1.444, 0.167) | 0.120 |
| **Log(MIP-1)** | N=23 |  | N=22 |  |  |  |
| Baseline | 2.241 | 1.500 | 1.450 | 2.149 |  |  |
| Return to ICU | 3.085 | 1.015 | 2.233 | 1.640 | -0.658 (-1.473, 0.156) | 0.110 |
| 6-12 hours | 2.797 | 1.494 | 1.814 | 1.906 | -0.678 (-1.701, 0.345) | 0.188 |
| 24 hours | 2.357 | 1.856 | 1.436 | 2.098 | -0.256 (-1.040, 0.529) | 0.514 |
| 48 hours | 2.281 | 1.650 | 1.672 | 2.098 | -0.118 (-1.022, 0.786) | 0.798 |
| Overall Effect |  |  |  |  | -0.440 (-1.085, 0.205) | 0.182 |

* Serum Il8 values were missing for 8 patients for a total of 13 observations.

†Per protocol analysis as specified in the statistical analysis plan included 49 patients (26 control, 23 washing)

# eTable 7. Organ Injury

|  | **Randomised patients** | | | |  |  |
| --- | --- | --- | --- | --- | --- | --- |
|  | **Standard care (N=29)** | | **Red cell washing (N=26)** | |  |  |
|  | **Mean** | **SD** | **Mean** | **SD** | **Effect (95% CI)** | **p-value** |
| **MODS Score Total** |  |  |  |  |  |  |
| Baseline | 0.32 | 0.61 | 0.69 | 1.23 |  |  |
| Return to ICU | 2.32 | 1.83 | 3.00 | 1.88 | 0.52 (-0.47, 1.51) | 0.293 |
| 6-12 hours | 1.96 | 1.75 | 2.50 | 1.45 | 0.42 (-0.43, 1.27) | 0.325 |
| 24 hours | 1.71 | 1.76 | 2.69 | 2.04 | 1.02 (-0.01, 2.04) | 0.051 |
| 48 hours | 2.07 | 2.39 | 2.92 | 2.24 | 0.67 (-0.50, 1.84) | 0.254 |
| 72 hours | 1.82 | 2.60 | 2.38 | 1.92 | 0.57 (-0.63, 1.78) | 0.341 |
| 120 hours | 1.25 | 2.59 | 1.42 | 1.30 | 0.11 (-1.02, 1.24) | 0.843 |
| Full Analysis Population  Overall effect |  |  |  |  | 0.56 (-0.26, 1.39) | 0.177 |
| **MODS Score maximum values** |  |  |  |  |  |  |
| **Worst value MODS** |  |  |  |  |  |  |
| Baseline | 0.31 | 0.60 | 0.67 | 1.21 |  |  |
| Sum of worst values | 4.41 | 3.13 | 5.37 | 2.57 | 0.73 (- 0.65, 2.11) | 0.296 |
| **Log(Serum Troponin)** |  |  |  |  |  |  |
| Baseline | 3.074 | 0.843 | 3.474 | 1.369 |  |  |
| Return to ICU | 8.731 | 1.029 | 8.520 | 1.014 | -0.183 (-0.734, 0.368) | 0.516 |
| 6-12 hours | 8.900 | 1.156 | 8.737 | 0.873 | -0.129 (-0.683, 0.426) | 0.650 |
| 24 hours | 8.449 | 1.285 | 8.251 | 0.809 | -0.151 (-0.733, 0.430) | 0.610 |
| 48 hours | 7.801 | 1.479 | 7.659 | 0.961 | -0.096 (-0.766, 0.573) | 0.778 |
| 72 hours | 7.306 | 1.481 | 7.065 | 0.892 | -0.185 (-0.848, 0.478) | 0.584 |
| 96 hours | 6.784 | 1.429 | 6.614 | 0.873 | -0.111 (-0.754, 0.532) | 0.736 |
| Overall effect |  |  |  |  | -0.175 (-0.705, 0.354) | 0.516 |
| **Log(Urine NGAL)** |  |  |  |  |  |  |
| Baseline | 2.006 | 1.240 | 1.433 | 1.031 |  |  |
| 6 hours | 3.374 | 0.699 | 3.446 | 0.773 | 0.161 (-0.254, 0.576) | 0.446 |
| 12 hours | 2.861 | 1.227 | 3.346 | 0.748 | 0.515 (-0.072, 1.101) | 0.086 |
| 24 hours | 2.576 | 0.928 | 3.119 | 0.684 | 0.496 (-0.017, 1.101) | 0.058 |
| Overall effect |  |  |  |  | 0.435 (0.022, 0.849) | 0.039 |
| Overall effect adjusted for baseline eGFR |  |  |  |  | 0.217 (-0.202, 0.636) | 0.311 |
| **Calculated log(creatinine clearance)** |  |  |  |  |  |  |
| 6 hours | 4.147 | 0.778 | 3.850 | 0.578 | -0.331 (-0.717, 0.054) | 0.092 |
| 12 hours | 4.318 | 0.770 | 3.829 | 0.727 | -0.531 (-0.962, -0.100) | 0.016 |
| 24-48 hours | 4.189 | 1.063 | 3.750 | 0.878 | -0.473 (-0.999, 0.054) | 0.078 |
| Overall effect |  |  |  |  | -0.445 (-0.793, -0.097) | 0.012 |
| Overall effect adjusted for baseline eGFR |  |  |  |  | -0.243 (-0.584, 0.097) | 0.161 |
| **Log (Serum Creatinine)** |  |  |  |  |  |  |
| Baseline | 4.31 | 0.32 | 4.57 | 0.34 |  |  |
| Return to ICU | 4.39 | 0.32 | 4.56 | 0.45 | -0.05 (-0.18, 0.08) | 0.474 |
| 6-12 hours | 4.52 | 0.34 | 4.75 | 0.37 | -0.03 (-0.13, 0.08) | 0.611 |
| 24 hours | 4.57 | 0.38 | 4.84 | 0.41 | 0.04 (-0.09, 0.18) | 0.537 |
| 48 hours | 4.61 | 0.45 | 4.93 | 0.55 | 0.02 (-0.16, 0.20) | 0.862 |
| 72 hours | 4.48 | 0.47 | 4.83 | 0.59 | 0.07 (-0.14, 0.28) | 0.503 |
| 120 hours | 4.45 | 0.44 | 4.75 | 0.56 | 0.02 (-0.19, 0.22) | 0.873 |
| Overall effect |  |  |  |  | -0.02 (-0.15, 0.12) | 0.809 |
| **paO2/FiO2 ratio** |  |  |  |  |  |  |
| Baseline | 482.05 | 108.31 | 484.27 | 164.26 |  |  |
| Return to ICU | 292.55 | 98.67 | 277.06 | 100.94 | -14.63 (-68.15, 38.90) | 0.585 |
| 6-12 hours | 345.89 | 111.73 | 340.84 | 97.75 | -6.24 (-61.55, 49.07) | 0.822 |
| 24 hours | 354.86 | 122.56 | 323.04 | 79.82 | -20.73 (-79.18, 37.72) | 0.479 |
| 48 hours | 328.32 | 115.74 | 325.91 | 112.62 | -7.69 (-64.21, 48.83) | 0.785 |
| 72 hours | 339.77 | 104.53 | 358.10 | 139.20 | 18.57 (-46.09, 83.22) | 0.566 |
| 120 hours | 411.64 | 139.19 | 384.05 | 102.08 | -28.53 (-92.91, 35.85) | 0.377 |
| Overall effect |  |  |  |  | -9.95 (-50.30, 30.40) | 0.622 |

# eTable 8: Secondary clinical outcomes

|  | **Randomised patients** | | | |  |  |
| --- | --- | --- | --- | --- | --- | --- |
|  | **Standard care (N=29)** | | **Red cell washing (N=27)** | |  |  |
| **CLINICAL OUTCOMES** | **N** | **%** | **n** | **%** | **Effect (95% CI)** | **p-value** |
| Organ injury, sepsis or death | 23 | 79.3% | 25 | 92.6% | 3.26* (0.58, 18.21) | 0.179 |
| Sepsis | 13 | 44.8% | 14 | 51.9% | 1.26* (0.43, 3.70) | 0.677 |
| Low cardiac output | 2 | 6.9% | 3 | 11.1% | ‡ |  |
| Acute kidney injury (Any) | 15 | 51.7% | 20 | 74.1% | 2.70 * (0.83, 8.85) | 0.100 |
| AKI Stage 1 | 11 | 37.9% | 12 | 44.4% |  |  |
| AKI Stage 2 | 2 | 6.9% | 4 | 14.8% |  |  |
| AKI Stage 3 | 2 | 6.9% | 4 | 14.8% |  |  |
| Overall |  |  |  |  | 1.68* (1.01, 2.82) | 0.048 |
| Adjusted for baseline eGFR |  |  |  |  | 1.37* (0.80, 2.36) | 0.254 |
| Berlin ARDS (Any) | 17 | 58.6% | 18 | 66.7% | 1.57* (0.48, 5.14) | 0.456 |
| Mild ARDS | 12 | 41.4% | 12 | 44.4% |  |  |
| Moderate ARDS | 5 | 17.2% | 4 | 14.8% |  |  |
| Severe ARDS | 0 | 0.0% | 0 | 0.0% |  |  |
| Stroke | 0 | 0.0% | 1 | 3.7% | ‡ |  |
| STEMI | 1 | 3.4% | 0 | 0.0% | ‡ |  |
| Death within 30 days of surgery | 1 | 3.4% | 0 | 0.0% | ‡ |  |
| **LENGTH OF STAY (median, IQR)** | |  |  |  |  |  |
| Ventilation time (hours) | 10.3 | (8.8, 16.2) | 11.2 | (8.6, 27.7) | 1.50† (0.85, 2.64) | 0.162 |
| CICU length of stay (hours) | 52.4 | (24.9, 144.8) | 115.1 | (48.2, 147.6) | 1.12† (0.65, 1.93) | 0.696 |
| Cardiac unit length of stay (days) | 11.1 | (9.0, 24.2) | 14.2 | (9.1, 22.0) | 0.93† (0.53, 1.61) | 0.783 |
| **EQ5D VAS summary index** |  |  |  |  |  |  |
| Pre-operative§ | 0.70 | 0.21 | 0.81 | 0.18 |  |  |
| 6-12 hours | 0.72 | 0.23 | 0.75 | 0.15 | -0.02 (-0.12, 0.07) | 0.621 |
| 6 weeks post-operative | 0.77 | 0.20 | 0.77 | 0.16 | 0.02 (-0.08, 0.12) | 0.683 |
| 3 months post-operative | 0.73 | 0.20 | 0.76 | 0.15 | 0.04 (-0.06, 0.14) | 0.433 |
| Overall treatment effect |  |  |  |  | 0.01** (-0.05, 0.08) | 0.685 |

*Odds ratio

†Hazard ratio

**Adjusted mean

‡ Analysis not done due to small number of observations

# eTable 9. Adverse events in the safety population

|  | **Received standard care (N=26)** | | | | **Received washed blood (N=26)** | | | |
| --- | --- | --- | --- | --- | --- | --- | --- | --- |
|  | **Adverse events** | | **SAEs** | | **Adverse events** | | **SAEs** | |
|  | **Events/ patients** | **%** | **Events/ patients** | **%** | **Events/ patients** | **%** | **Events/ patients** | **%** |
| **EXPECTED EVENTS (I.E. LISTED IN THE STUDY PROTOCOL)** | | | |  |  |  |  |  |
| STEMI | 1 | 3.8% | 0 | 0.0% | 0 | 0.0% | 0 | 0.0% |
| Cardiac arrest | 0 | 0.0% | 0 | 0.0% | 0 | 0.0% | 0 | 0.0% |
| SVT/AF | 14 | 53.8% | 0 | 0.0% | 9 | 34.6% | 1 | 3.8% |
| VF/VT | 0 | 0.0% | 0 | 0.0% | 1 | 3.8% | 0 | 0.0% |
| New pacing | 4 | 15.4% | 0 | 0.0% | 7 | 26.9% | 0 | 0.0% |
| Use of inotropes | 24 | 92.3% | 1 | 3.8% | 25 | 96.2% | 2 |  |
| Use of intraaortic balloon pump | 1 | 3.8% | 0 | 0.0% | 0 | 0.0% | 0 | 0.0% |
| Use of pulmonary artery catheter | 9 | 34.6% | 0 | 0.0% | 7 | 26.9% | 0 | 0.0% |
| Use of vasodilator | 4 | 15.4% | 0 | 0.0% | 4 | 15.4% | 0 | 0.0% |
| Low cardiac output | 2 | 7.7% | 0 | 0.0% | 3 | 11.5% | 0 | 0.0% |
| Tracheostomy | 1 | 3.8% | 1 | 3.8% | 0 | 0.0% | 0 | 0.0% |
| Mask CPAP | 1 | 3.8% | 0 | 0.0% | 5 | 19.2% | 1 | 3.8% |
| Pneumothorax or effusion requiring draining | 3 | 11.5% | 1 | 3.8% | 7 | 26.9% | 1 | 3.8% |
| Acute kidney injury | 15 | 57.7% | 0 | 0.0% | 19 | 73.1% | 1 | 3.8% |
| AKIN stage 1 | 11 | 42.3% | 0 | 0.0% | 11 | 42.3% | 0 | 0.0% |
| AKIN stage 2 | 2 | 7.7% | 0 | 0.0% | 4 | 15.4% | 0 | 0.0% |
| AKIN stage 3 | 2 | 7.7% | 0 | 0.0% | 4 | 15.4% | 1 | 3.8% |
| Haemofiltration/dialysis since heart operation | 1 | 3.8% | 0 | 0.0% | 3 | 11.5% | 1 | 3.8% |
| Peptic ulcer/ GI bleed/ perforation◦ | 1 | 3.8% | 1 | 3.8% | 1 | 3.8% | 1 | 3.8% |
| Other GI | 0 | 0.0% | 0 | 0.0% | 1 | 3.8% | 0 | 0.0% |
| Permanent stroke | 0 | 0.0% | 0 | 0.0% | 1 | 3.8% | 1 | 3.8% |
| TIA | 0 | 0.0% | 0 | 0.0% | 0 | 0.0% | 0 | 0.0% |
| Excessive bleeding not requiring re-operation | 0 | 0.0% | 0 | 0.0% | 0 | 0.0% | 0 | 0.0% |
| Wound dehiscence | 2 | 7.7% | 1 | 3.8% | 0 | 0.0% | 0 | 0.0% |
| Reoperation | 0 | 0.0% | 0 | 0.0% | 0 | 0.0% | 0 | 0.0% |
|  | | | | | |  |  |  |

# Changes to the REDWASH Trial after trial commencement.

| **Amendment number**  **(i.e. REC and/or MHRA amendment number)** | **Previous version** | **Previous date** | **New version** | **New date** | **Brief summary of changes** |
| --- | --- | --- | --- | --- | --- |
| 1 | 1.0 | 26/10/2012 | 2.0 | 22/04/2013 | Revised Health Economic analysis plan  Addition of EQ5D and resource use questionnaire at 6 weeks and 3 months post-randomisation  Change of definition of end of study (to completion of health economic assessment at 3 months post-randomisation)  Description of data collection procedures for point 2 above  Addition of relevant references |
| 2 | 2.0 | 22/04/2013 | 3.0 | 04/08/2013 | Removal of upper age limit for study participants (inclusion criterion 1; 80 years)  Specification of 25 percentage risk for massive blood transfusion (inclusion criterion 2)  Removal of ROTEM test for Glenfield participants  Minor administrative changes |
| 3 | 3.0 | 04/08/2013 | 4.0 | 16/09/2013 | Removal exclusion criterion 5: Patients with a pre-existing inflammatory state (e.g. sepsis, active inflammatory disease including active rheumatoid arthritis, colitis, Lupus, or Crohn’s disease. NB – consider latter conditions as active conditions when a patient is taking a high dose of oral steroids, for example > 10 mg per day of prednisolone)."  Addition of interim evaluation of experimental markers of inflammation and organ injury in first 60 patients at Glenfield Hospital |
| SAP |  |  |  |  | The study was been stopped prematurely by the funder because of slow recruitment. The final analysis will now take place, with 60 consented patients.  The primary outcome was confirmed as serum cytokine IL-8 with repeated measures at four post-surgery time points: on return to ITU, and 6-12 hours, 24 and 48 hours post-operatively.  ARDS was redefined according to the Berlin ARDS definition [Ranieri, 2012] as PaO2/FiO2 ratio < 300 mmHg where the Continuous Positive Airway Pressure (CPAP) or Positive End Expiratory Pressure (PEEP) is > 5cmH2O.  Blood loss was recorded at 4 hours and 12 hours, rather than the 6 hours given in the protocol.  The study did not measure GM-CSF, IFN-γ, IL-1β, IL-2, IL-4, IL-5, and IL-10, as stated in the protocol. Levels of IL-6 and TNF- α were recorded as planned and MIP-1 and MCP-1 were recorded in addition. |

# Statistical Analysis Plan

**A Randomised Controlled Trial of Red Cell Washing for the Attenuation of Transfusion Associated Organ Injury in Cardiac Surgery**

**(REDWASH)**

**SAP Version:**  **0.8**

**Date:**  13-Jul-2015

**Based on protocol / protocol amendment :** REDWASH-00

**Version:** *3.0*

**Date:** *04-Aug-2013*

Sponsor Name: University of Leicester

Sponsor Address: Research Support Office

University Road

Leicester, LE1 7RH

Funder : NIHR

Trial Statistician: Tom Morris

Medical Statistician

University of Leicester

College of Medicine, Biological Sciences and Psychology

Leicester Diabetes Centre, Leicester General Hospital

Leicester LE5 4PW

Chief Investigator: Mr Gavin Murphy

Professor of Cardiac Surgery

University of Leicester

Glenfield Hospital, Groby Road

Leicester, LE3 9QP

**Trial registration**

MREC No 12/EM/0475

ISRCTN 27076315

UKCRN ID 13922

**Revision History (only circulated versions)**

| Version | Date | Author(s) and Role | Summary of Changes/Comments |
| --- | --- | --- | --- |
| 0.1 | 12-MAY-2015 | Tom Morris  (Trial Statistician) | Initial version |
| 0.3 | 29-MAY-2015 | Tom Morris  Gavin Murphy | Inclusion of GM comments following SAP meeting on 20/05/2015. |
| 0.6 | 10-JUN-2015 | Tom Morris  Gavin Murphy | Further comments, and corrections to time points. |
| 0.8 | 13-JUL-2015 | Tom Morris  Gavin Murphy  Cassey Brookes | Inclusion of CB comments. |
|  |  |  |  |

**SAP approval for finalised versions:**

| **Lead Statistician** |  | *Tom Morris* |  |  |
| --- | --- | --- | --- | --- |
|  |  |  |  |  |
|  |  |  |  |  |
|  |  | Signature |  | Date |

| **Principal Investigator** |  | *Gavin Murphy* |  |  |
| --- | --- | --- | --- | --- |
|  |  |  |  |  |
|  |  |  |  |  |
|  |  | Signature |  | Date |
| **Supervising Statistician** |  | *Cassey Brookes* |  |  |
|  |  |  |  |  |
|  |  |  |  |  |
|  |  | Signature |  | Date |

LIST OF ABBREVIATIONS

| AE | Adverse Event |
| --- | --- |
| AKI | Acute Kidney Injury |
| ARDS | Acute respiratory distress syndrome |
| BRU | Biomedical Research Unit |
| CI | Chief Investigator |
| CPB | Cardiopulmonary Bypass |
| CRF | Case Report Form |
| CTP | Clinical Trial Protocol |
| CTR | Clinical Trial Report |
| CTU | Clinical Trials Unit |
| FDA | Food and Drug Administration |
| GM-CSF | Granulocyte macrophage colony stimulating factor |
| HDU | High Dependency Unit |
| ICH | International conference of Harmonisation |
| ICU | Intensive Care Unit |
| IFN-γ | Interferon gamma |
| IL | Interleukin |
| ITT | Intention-to-treat |
| ITU | Intensive Therapy Unit |
| KDIGO | International Kidney Diseases: Improving Global Outcomes |
| LFABP | Renal liver-type fatty acid binding protein |
| MI | Myocardial infarction |
| MODS | Multiple Organ Dysfunction Score |
| NGAL | Neutrophil gelatinase-associated lipocalin |
| NHSBT | NHS Blood and Transplant |
| PPS | Per protocol set |
| RBC | Red Blood Cells |
| SAE | Serious Adverse Event |
| SAF | Safety population |
| SAP | Statistical Analysis Plan |
| SIRS | Systemic Inflammatory Response Syndrome |
| SUSAR | Suspected Unexpected Serious Adverse Reaction |
| TNF-α | Tumour necrosis factor alpha |

Contents

[1 Introduction 7](#_Toc404855596)

[1.1 Study Objectives 8](#_Toc404855597)

[1.1.1 Primary Objectives 8](#_Toc404855598)

[1.1.2 Secondary Objectives 8](#_Toc404855599)

[1.1.3 Exploratory Objectives 8](#_Toc404855600)

[1.2 Study Design 9](#_Toc404855601)

[1.2.1 Study Interventions 9](#_Toc404855602)

[1.2.2 Intervention Group 9](#_Toc404855603)

[1.2.3 Control Group 9](#_Toc404855604)

[1.2.4 Sample size 10](#_Toc404855605)

[1.3 Schedule of Major Assessments 11](#_Toc404855606)

[1.4 Changes of the Planned Analysis 12](#_Toc404855607)

[2 Outcomes 13](#_Toc404855608)

[2.1 Primary Outcomes 13](#_Toc404855609)

[2.1.1 Definition of Primary Outcomes 13](#_Toc404855610)

[2.1.2 Hypotheses to be investigated 13](#_Toc404855611)

[2.1.3 Handling of Missing Data 14](#_Toc404855612)

[2.2 Secondary Outcomes 14](#_Toc404855613)

[2.2.1 Definition and Derivation of Secondary Outcomes 14](#_Toc404855614)

[2.2.2 Hypotheses to be investigated 15](#_Toc404855615)

[2.2.3 Handling of Missing Data 15](#_Toc404855616)

[2.3 Other Efficacy Outcomes 15](#_Toc404855617)

[2.4 Other Outcomes of Interest 15](#_Toc404855618)

[3 Analysis Sets/Populations 16](#_Toc404855619)

[3.1 Protocol deviations (PD) 16](#_Toc404855620)

[3.2 Intent-to-treat Population / Full analysis set 16](#_Toc404855621)

[3.3 Per-protocol sets 16](#_Toc404855622)

[3.4 Safety Population 16](#_Toc404855623)

[3.5 Other Analysis Populations 16](#_Toc404855624)

[4 General Issues for Statistical Analysis 17](#_Toc404855625)

[4.1 Derived/ Computed Variables 17](#_Toc404855626)

[4.2 Multiplicity, Multiple Comparisons and Interim Analyses 17](#_Toc404855627)

[4.3 Planned Subgroups, Interactions and Covariates 17](#_Toc404855628)

[4.4 Analysis Software 18](#_Toc404855629)

[5 Statistical Methodology 19](#_Toc404855630)

[5.1 Disposition of Patients 19](#_Toc404855631)

[5.2 Demographic and Baseline Characteristics 19](#_Toc404855632)

[5.3 Primary Outcome Analysis 19](#_Toc404855633)

[5.3.1 Primary Analysis of Primary Outcomes 19](#_Toc404855634)

[5.3.2 Secondary Analyses of Primary Outcomes 20](#_Toc404855635)

[5.3.3 Sensitivity and Exploratory Analyses 20](#_Toc404855636)

[5.3.4 Subgroup Analyses 20](#_Toc404855637)

[5.4 Secondary Outcome Analyses 20](#_Toc404855638)

[5.5 Additional Efficacy Analyses 21](#_Toc404855639)

[6 Safety and Tolerability Analysis 22](#_Toc404855640)

[6.1 Drug Exposure 22](#_Toc404855641)

[6.2 Adverse Events and Tolerability 22](#_Toc404855642)

[6.3 Laboratory Data 23](#_Toc404855643)

[6.4 Vital Signs, Electrocardiogram and Other Safety Assessments 23](#_Toc404855644)

[7 References 24](#_Toc404855645)

[8 Appendices 26](#_Toc404855646)

[8.1 Publication strategy 26](#_Toc404855647)

[8.2 Roles in the analysis 26](#_Toc404855648)

[8.3 Technical specification 26](#_Toc404855649)

# Introduction

This Statistical Analysis Plan (SAP) describes the planned analysis and reporting for the trial Murphy_12_130 REDWASH (protocol REDWASH-001). The structure and content of this SAP provides sufficient detail to meet the requirements identified by the FDA and International Conference on Harmonization of Technical Requirements for Registration of Pharmaceuticals for Human Use (ICH): Guidance on Statistical Principles in Clinical Trials. All work planned and reported for this SAP will follow internationally accepted guidelines, published by the American Statistical Association and the Royal Statistical Society for statistical practice.

The reader of this SAP is encouraged also to read the clinical protocol for details on the conduct of this study, and the operational aspects of clinical assessments and timing for the process of completing a patient in this study.

The purpose of this SAP is to outline the planned analyses that are to be performed on the data to support the completion of the Clinical Trial Report (CTR). The SAP will be amended if there are substantial changes to the planned analyses, and in any case, will be finalized before the database lock for this study. Exploratory post-hoc or unplanned analyses not necessarily identified in this SAP may be performed on these data as required. These analyses will be clearly identified in the CTR.

**Throughout the document: Any verbatim text from the protocol is provided in italics inside a border:**

*Text from the protocol.*

## Study Objectives

*The REDWASH Trial proposes to test the hypothesis that that the severity of the postoperative inflammatory response will be less and post-operative recovery faster if patients undergoing cardiac surgery with CPB who are at risk of massive RBC transfusion receive stored allogenic RBC that are washed prior to transfusion when compared to standard care where stored RBC are administered without washing.*

### Primary Objectives

*A. Estimate mean differences in biochemical markers of the systemic inflammatory response between participants allocated to receive washed versus unwashed RBC.*

### Secondary Objectives

*A secondary hypothesis is that the adverse effects of transfusion are mediated by platelet and monocyte activation by microparticles within the storage supernatant and that by removing the supernatant this is attenuated.*

…

*B. Estimate mean differences in hospital length of stay between participants allocated to receive washed versus unwashed RBC.*

*C. Estimate differences in the frequency of inflammatory organ injury or death between participants allocated to receive washed versus unwashed RBC.*

*D. Estimate the cost-effectiveness of washed versus unwashed RBC.*

*E. Establish whether red cell washing attenuates postoperative platelet and monocyte activation (subgroup analysis).*

### Exploratory Objectives

None.

## Study Design

*This study is a multi-centre, randomised, single blinded parallel group, randomised controlled trial of washing of allogenic RBC prior to transfusion versus standard care (no washing).*

### Study Interventions

*Eligible patients undergoing cardiac surgery with CPB who consent to participate will be randomly allocated, in a 1:1 ratio to:*

*1. GROUP A: Unwashed RBC (standard care)*

*2. GROUP B: Washed RBC*

### Intervention Group

*Allogenic RBC, harvested in citrate-adenine-phosphate-dextrose, buffy coat removed, leucocyte depleted, saline-adenine-glucose-mannitol stored red cell units, supplied by NHSBT as per standard practice will be used. For the intervention each unit of RBC will be added to a Continuous AutoTransfusion System, washed using a centrifugal method, as per the device instructions. The washed RBC will then be immediately administered to the patient as per standard practice.*

### Control Group

*Allogenic RBC, harvested in citrate-adenine-phosphate-dextrose, buffy coat removed, leucocyte depleted, saline-adenine-glucose-mannitol stored red cell units, supplied by NHSBT as per standard practice will be used.*

### Sample size

*The primary outcome, serum IL8 levels, is continuously scaled, so the target differences can be specified as a “standardised differences” (0.2=small, 0.5=moderate, 0.8=large). On the assumption that there will be a moderate correlation of 0.7 between pre and post-intervention measures and between repeated post-intervention measures, as observed in previous work [35], and on the basis that there will be one baseline and three postoperative measures, we estimate that a sample size of 150 patients will allow us to detect a small to moderate target difference between groups of 0.4, with 90% power and 5% significance (2-tailed). We propose to recruit 170 patients (85 per group) assuming an attrition rate of between 10-15% for incomplete sampling, patient death and withdrawal. The sample size will also allow us to detect a 40% difference in the composite endpoint of any sepsis, inflammatory organ injury or death with 80% power and 5% significance. For time to ICU / hospital discharge, the sample size will allow a hazard ratio of 1.65 to be detected.*

### Blinding

*…the investigators, including those responsible for the collection of postoperative data and laboratory and statistical analyses will be blinded.*

The analysis will initially be conducted using masked treatment groups and with data that could lead to unblinding removed (blood data, adverse events). Once this initial analysis is complete, the treatment groups will be revealed and the remaining data added to the analysis.

## Schedule of Major Assessments

|  | Pre-Op’n | Op’n Day | Day 1 | Day 2 | Day 3 | Day 4 | Dis-charge | 6 wks | 3 months |
| --- | --- | --- | --- | --- | --- | --- | --- | --- | --- |
| Eligibility | ✓ |  |  |  |  |  |  |  |  |
| Written consent | ✓ |  |  |  |  |  |  |  |  |
| Randomisation | ✓ |  |  |  |  |  |  |  |  |
| EQ5D Questionnaire | ✓ |  |  |  |  |  | ✓ | ✓ | ✓Ʊ |
| Bloods: serum biochemistry (creatinine and troponin T/I) | ✓ † | ✓ †  (CICU & 6 -12 hrs) | ✓ †  (24 hrs) | ✓ †  (48 hrs) | ✓ †  (72 hrs) | ✓λ  (96 hrs) | ✓ λ |  |  |
| Bloods: serum inflammatory biomarkers | ✓ † | ✓ †  (CICU & 6 -12 hrs) | ✓ †  (24 hrs) | ✓ †  (48 hrs) |  |  |  |  |  |
| Bloods: Full blood counts | ✓ | ✓  (CICU & 6 -12 hrs) | ✓  (24 hrs) | ✓  (48 hrs) | ✓  (72 hrs) | ✓λ  (96 hrs) | ✓ λ |  |  |
| Bloods: Plasma sample for MP analysis and monocyte activation | ✓ | ✓  (CICU) | ✓  (24 hrs) | ✓  (48 hrs) |  |  |  |  |  |
| Urine sample & volume: NGAL, urea and elecrolytes | ✓ | ✓  (6 & 12 hrs) | ✓  (24 hrs) | ✓ † α  (48 hrs) |  |  |  |  |  |
| Operative details |  | ✓ † |  |  |  |  |  |  |  |
| Clinical outcomes |  |  |  |  |  | ✓ | ✓ | ✓§ |  |
| Serious adverse event monitoring |  | ✓ | ✓ | ✓ | ✓ | ✓ | ✓ | ✓§ |  |
| Resource use data |  |  |  |  |  |  | ✓ | ✓§ | ✓Ʊ |
| Bloods: Platelet response ¥ | ✓ ¥ | ✓¥  (CICU & 6 -12 hrs) | ✓ ¥  (24 hrs) | ✓ ¥  (48 hrs) |  |  |  |  |  |

Λ Discharge time point if hospital stay exceeds 5 days

§ 4-6 week time point in accordance with normal postoperative care.

† Indicates samples taken as part of normal care.

α Indicates sample for determination of routine urea and electrolytes only

¥ Indicates Glenfield patients alone

Ʊ Indicates data collection via postal questionnaire

## Changes to the Planned Analysis

The study has been stopped prematurely by the funder because of slow recruitment. The final analysis will now take place, with 60 consented patients.

The primary outcome is confirmed as serum cytokine IL-8 with repeated measures at four post-surgery time points: on return to ITU, and 6-12 hours, 24 and 48 hours post-operatively.

ARDS is now defined according to the Berlin definition [Ranieri, 2012] as PaO2/FiO2 ratio < 300 mmHg where the Continuous Positive Airway Pressure (CPAP) or Positive End Expiratory Pressure (PEEP) is > 5cmH2O.

Blood loss is recorded at 4 hours and 12 hours, rather than the 6 hours given in the protocol.

The study did not measure GM-CSF, IFN-γ, IL-1β, IL-2, IL-4, IL-5, and IL-10, as stated in the protocol. Levels of IL-6 and TNF- α were recorded as planned and MIP-1 and MCP-1 were recorded in addition.

# Outcomes

A complete list of variables to be evaluated in the statistical analysis is provided in a separate document, “REDWASH outcomes v0.3”.

## Primary Outcomes

### Definition of Primary Outcomes

*The primary outcome for the trial is the severity of the systemic inflammatory response as indicated by serum cytokine levels IL8. These will be measured from venous blood samples taken preoperatively, on return to ITU, and 6 hours, 24, 48 and 96 hours post-operatively.*

### Hypotheses to be investigated

The primary hypothesis is that serum cytokine IL-8 will be reduced by transfusion with washed red blood cells in comparison to unwashed red blood cells over the first 96 hours post-operatively.

The difference in serum cytokine IL-8 will also be investigated at four time points individually, namely return to ITU, 6-12, 24 and 48 hours post-operatively.

### Handling of Missing Data

*Any missing outcome and cost data will be dealt with using multiple imputation methods.*

Lab data below the limit of quantification will be imputed as half the limit of quantification.

## Secondary Outcomes

### Definition and Derivation of Secondary Outcomes

*In addition we will measure serum levels of GM-CSF, IFN-γ, IL-1β, IL-2, IL-4, IL-5, IL-6, MCP-1, MIP-1 IL-10 and TNF-α.*

Serum levels of MIP-1 and MCP-1 were measured in place of GM-CSF, IFN-γ, IL-1β, IL-2, IL-4, IL-5 and IL-10. Serum levels of IL-6 and TNF-α were recorded as planned

(a) *Inflammatory Organ Injury, Sepsis or Death*:

- *Sepsis will be defined as Antibiotic treatment for suspected infection,* ***and*** *the presence of SIRS within 24 hours prior to start of antibiotic treatment where SIRS is defined as ≥ 2 of the following conditions: temperature > 38 ^o^C or < 36 ^o^C; heart rate > 90 beats per min; respiratory rate > 20 breaths per min or PaCO_2_ < 32 mmHg; white blood cell count > 12,000 per mm^3^ or < 4,000 per mm^3^,* ***or*** *antibiotic treatment for wound infection.*
- *Acute Kidney Injury, defined as KDIGO stage 1, 2 or 3.*
- *Acute lung injury, defined as PaO_2_/FiO_2_ ratio <300mmHg or a requirement for respiratory support; invasive ventilation >48 hours, non-invasive ventilation >4 hours, reintubation, tracheostomy, or ARDS.*
- *Low cardiac output, defined as new intra-or postoperative intra-aortic balloon pump insertion or a cardiac index of <2.2 L · min^−1^ · m^−2^ refractory to appropriate intravascular volume expansion after correction or attempted correction of any dysrhythmias, or the administration of ionotropes including Dobutamine, Enoximone, Milrinone, Levosimendan and Adrenaline.*
- *Death*
- *Differences in Multiple Organ Dysfunction Score [36] at days 1, 2, 3 and 5.*

Sepsis is defined as at least one of the following:

- antibiotic treatment for suspected infection AND presence of SIRS
- antibiotic treatment for wound infection.

Since the publication of the protocol, a new consensus definition (the Berlin definition) of ARDS has been published [Ranieri, 2012]. This replaces both the old definition of ARDS and the term “acute lung injury”, which has been abandoned. ARDS is now defined as PaO_2_/FiO_2_ ratio <300 mmHg where the Continuous Positive Airway Pressure (CPAP) or Positive End Expiratory Pressure (PEEP) is > 5cmH2O. ARDS will be defined using the Berlin criteria as Mild, Moderate or Severe.

Due to this change, the outcome “requirement for respiratory support” will not be reported.

The inotropes included in the definition of low cardiac output are an exhaustive list.

Each of the six components of the Multiple Organ Dysfunction Score (MODS) will be an outcome, as well as forming the overall MODS outcome. See section 4.1 for the derivation of the MODS.

*The sample size will allow us to detect a 40% difference in the composite endpoint of any sepsis, inflammatory organ injury or death…*

A composite outcome consisting of sepsis, acute kidney injury, ARDS, low cardiac output and death will be derived.

(b) *Bleeding and Transfusion*

- *Blood loss at 6 hours postoperatively.*
- *The number of units of RBC and other blood components transfused during the operative period and post-operative hospital stay will be recorded*

Blood loss is recorded at 4 hours and 12 hours, rather than the 6 hours given in the protocol.

(c) *Transfusion Reactions*

- *Febrile Transfusion Reactions*
- *Non-haemolytic transfusion reactions.*
- *Haemolytic Transfusion reactions.*

The transfusion reactions are potential adverse events and will be recorded only on the adverse event log. Since these will be entered as free-text, the Chief Investigator will confirm which adverse events can be categorised as one of these reactions on a case-by-case basis.

(d) *Other clinical outcomes*

- *Stroke; diagnosed by brain imaging (CT or MRI), in association with new onset focal or generalised neurological deficit (defined as deficit in motor, sensory or co-ordination functions)*
- *ST elevation myocardial infarction accompanied by troponin I > 5000 pg ml^-1^*

(e) *Hospital stay and cumulative resource use*

- *ITU, HDU and hospital length of stay will be determined by the assessment of care level (see section 5.12 and reference 37).*
- *Resource use will be costed using credible nationally published sources.*
- *An incremental cost per complication avoided will be calculated (further details in section 6.4).*

The following time-to-event outcomes will be evaluated:

- ventilation time / time to extubation
- time to discharge from CICU/HDU
- time to discharge from hospital

Ventilation time is defined as the time between return from theatre and extubation.

Time to discharge from CICU/HDU is defined as the sum of the time between admission to CICU/HDU and admission to ward, and the time between return to CICU/HDU and return to ward. Note that if there was no return to CICU/HDU then this second time period is 0.

Time to discharge from hospital is defined as the time from return from theatre to discharge.

All time are measured in hours.

No resource use or cost analyses will be conducted due to the early termination of the study. The EQ-5D was recorded to be part of the intended health economic analyses, and this will now be presented instead as part of the statistical analysis.

(f) *Compliance with the washing protocol*

- *Data will be collected for all patients during surgery to characterise compliance with the randomly assigned washing protocol.*

(g) *Additional markers of inflammation and organ injury*

- *Urinary Liver Fatty Acid Binding Protein (LFABP), Neutrophil gelatinase associated lipocalin (NGAL) at baseline and at 6, 12 and 24 hours for a* *sub-group of patients (n= 40 per group).*
- *Serum Troponin I at baseline and at 24 and 48 hours.*
- *Platelet aggregation (Multiplate®) in the first 48hrs for a sub-group of patients (patients at Glenfield hospital only).*
- *Age of each unit of RBC transfused.*
- *Transfused RBC characteristics (washed and unwashed); Adenosine Triphosphate (ATP) levels, 2,3DPG, deformability, osmotic fragility, cytokine levels.*
- *Platelet and monocyte activation as determined by flow cytometry for a sub-group of patients (patients at Glenfield hospital only).*
- *Endothelial injury as determined by quantification of endothelial derived microparticles (as per figure 3) by flow cytometry.*
- *Effect of blood harvested from recipients on platelet and monocyte activation within a microfluidics system, as per figure 5)*

Due to the lack of funding, the following outcomes were not recorded:

- LFABP
- transfused RBC characteristics (except age of each unit)
- effect of blood on platelet and monocyte activation

NGAL was measured in all patients, not a subset.

Serum troponin I was measured at baseline and on return to ITU, 6-12, 24, 48, 72 and 96 hours post-operatively.

Platelet aggregation was measured at baseline and on return to ITU, 6-12, 24 and 48 hours. This outcome, together with endothelial injury, will be analysed in an exploratory fashion separate to the clinical trial analyses. The results from these external analyses will be presented together with the clinical trial results in a joint publication. See section 8.1.

Creatinine clearance will be an additional outcome, calculated as in section 4.1.

### Hypotheses to be investigated

All secondary outcomes are hypothesised to benefit from the intervention.

### Handling of Missing Data

*Any missing outcome and cost data will be dealt with using multiple imputation methods.*

Lab data below the limit of quantification will be imputed as half the limit of quantification.

When individual components of the MODS are missing, imputation rules are built in to the definition of the score.

## Other Efficacy Outcomes

None.

## Other Outcomes of Interest

Compliance with the washing protocol.

### Handling of Missing Data

If historic dates are not fully known (e.g. medical history), the following rules for data entry will be applied at data entry:

- If Month and Year are known, but not the exact date, the 15^th^ will be used as imputation rule.
- If only the Year is known, the 01-Jul will be used as imputation rule.

# Analysis Sets/Populations

## Protocol deviations (PD)

Time window violations are generally not considered as major protocol deviations.

Patients who underwent randomisation but did not undergo surgery will not be considered in the analysis. Patients entered into the trial in error (found post randomisation and surgery to be ineligible) will be included in the safety population only.

Receipt of only unwashed blood for patients randomised to receive washed blood will be considered a major protocol violation.

Receipt of some units of unwashed blood in a patient randomised to receive washed blood, and who receive at least 1 unit of washed blood, will be considered a minor protocol violation. Patients not followed up to the end of the trial (3 months) will be considered minor protocol violations. Patients with missing data that relate to laboratory and biochemical values will also be considered as minor protocol violations.

## Intention-to-treat Population / Full analysis set (ITT/FAS)

*The trial will be analysed on an intention-to-treat basis.*

The intention-to-treat population comprises all patients randomised into the trial who underwent surgery, with patients considered to be in the group they were randomised to, regardless of the treatment they eventually received.

## Per-protocol sets (PPS)

The per-protocol population comprises all patients who have been recruited into the trial, had the trial intervention administered and who do not have major protocol deviations*.*

## Safety Population (SAF)

The safety population comprises all patients who have been recruited into the trial and had the trial intervention administered*.* In this population, patients are considered to be in the intervention group if they received at least one unit of washed blood. Otherwise, they are considered to be in the control group. The SAF will be used for all safety analyses.

## Other Analysis Populations

Not applicable.

# General Issues for Statistical Analysis

## Derived/ Computed Variables

Sepsis

Sepsis is defined as at least one of the following:

- antibiotic treatment for suspected infection AND presence of SIRS
- antibiotic treatment for wound infection

where SIRS is defined as at least two of the following:

- temperature > 38⁰C or < 36⁰C
- heart rate > 90bmp
- respiratory rate > 20 breaths per minute OR PaCO_2_ < 32mmHg
- white blood cell count > 12,000 mm^-3^ or < 4,000 mm^-3^

ARDS

ARDS is defined according to the new Berlin definition [Ranieri, 2012].

Creatinine clearance

Creatinine clearance will be calculated by the formula

$$CCr \left[ {ml}/{min} \right]=\frac{UCr \left[ {mmol}/l \right]\times Vdt \left[ {ml}/{min} \right]}{PCr {[\mu mol}/{l]} \times1000}$$

where Creatinine Clearance ($CCr$) is calculated from the creatinine concentration in the collected urine sample ($UCr$), urine flow rate ($Vdt$), and the plasma concentration ($PCr$).

MODS

The MODS comprises six components which are objectively scored from 0 to 4, then summed to form a total from 0 to 24, where higher scores indicate higher organ dysfunction. In this study it was recorded at baseline and at six time-points during follow-up, and will be analysed using two methods. The first will be a repeated measures analysis consisting of the MODS at each time point. The second will be a derived “worst value” MODS that was recently used in the RECESS trial [Steiner, 2015], defined as follows:

At time point $i=0,\ldots,6$ (where 0 is baseline) and MODS component $j=1,\ldots,6$ let $M_{ij}$ be the score of the component, from 0 to 4. Then the worst-value score for MODS component $j$ is

$${M^{*}}_{j}=\max\left\{ M_{ij} | i=1,\ldots,6 \right\}.$$

The overall worst-value MODS is then

$$MODS= \sum_{j=1}^{6} {M^{*}}_{j}$$

and the change from baseline is

$$\Delta MODS= \sum_{j=1}^{6} \left( {M^{*}}_{j}-M_{0j} \right).$$

Other continuous variables

In a similar manner, secondary analyses will be conducted on the continuous outcomes using “highest values” and “lowest values” over time. Specifically, for a continuous outcome $y_{i}$ measured at time points $i=1,\ldots,n$, the following outcomes will be derived:

$$max\left\{ y_{i} | i=1,\ldots,n \right\} \text{and} min\left\{ y_{i} | i=1,\ldots,n \right\}.$$

EQ-5D

For the EQ-5D-3L questionnaire, a summary score will be calculated at each time point for each patient. This will be based on the VAS index derivation [Euroqol].

|  | **VAS  value set UK** |
| --- | --- |
| Full health (11111) | 1 |
|  |  |
| At least one 2 or 3 (constant) | -0.155 |
| At least one 3 (N3) | -0.215 |
|  |  |
| Mobility = 2 | -0.071 |
| Mobility = 3 | -0.182 |
|  |  |
| Self care = 2 | -0.093 |
| Self care = 3 | -0.145 |
|  |  |
| Usual activities = 2 | -0.031 |
| Usual activities = 3 | -0.081 |
|  |  |
| Pain/discomfort = 2 | -0.084 |
| Pain/discomfort = 3 | -0.171 |
|  |  |
| Anxiety/depression = 2 | -0.063 |
| Anxiety/depression = 3 | -0.124 |

## Multiplicity, Multiple Comparisons and Interim Analyses

*The primary analysis will take place when follow-up is complete for all recruited patients. No formal interim analysis is planned. Outcome data will be reported to the Data Monitoring and Safety Committee every 6 months, together with any additional analyses the committee request. In these reports the data will be presented by group but the allocation will remain masked.*

The study has been stopped prematurely and the final analysis will now take place, with 60 patients having been recruited, of whom 56 have undergone surgery.

There will be no adjustment for multiple comparisons.

## Planned Subgroups, Interactions and Covariates

*Two sensitivity analyses are planned, comparing the primary outcome for … 2. Patients who receive older blood versus younger blood, i.e. those who receive only blood less than 14 days old versus those that receive any blood over 14 days old, on the basis that the proposed intervention is expected to prevent the risks attributed to prolonged blood storage.*

These subgroup analyses will not be undertaken due to the small number of patients for whom data were recorded.

## Analysis Software

The clinical data will be extracted from a MACRO data base. Laboratory data will be transferred from the lab sites to the CTU via validated EXCEL sheets.

The analysis will be performed with a current version of SAS™. Graphical displays will generally be generated using R, possibly using the graphics package “ggplot2”.

CRF Data without values might be coded as “not available” (by investigator) or “missing” (by data base). Any data which are derived from missing or not available data will however always be coded as “missing” only.

# Statistical Methodology

The statistical analysis will be based on external guidelines (e.g. ICH E3 and E9), and displayed in accordance with the CTU display catalogue and other external recommendations [Bamnote, 2012].

The table of contents of all analyses will be planned on an EXCEL table, including the risk assessment and the validation methods of each display.

## The date of data extraction from the database will be included in all tables and listings.Disposition of Patients

Patient disposition will be presented with respect to completion status, reason for non-completion and length of stay in trial. Results will be tabulated and summarised over time by treatment group and in total.

A CONSORT chart will display the flow of patients through the trial.

A graph of cumulative recruitment will be presented.

## Demographic and Baseline Characteristics

The demographic and clinical characteristics and the medical history and concomitant medication will be tabulated and summarised by treatment group and in total. This will include the stratification variables.

Numbers (with percentages) for binary and categorical variables and means (and standard deviations), and medians (with lower and upper quartiles) for continuous variables will be presented.

There will be no formal comparison of baseline variables between treatment groups.

## Primary Outcome Analysis

### Primary Analysis of Primary Outcomes

*The primary analysis will take place when follow-up is complete for all patients and will be performed on an intention-to-treat basis. Means for continuous outcomes (transformed logarithmically if required) will be compared using analysis of variance or regression modelling, adjusting for baseline values where available. Findings will be reported as effect sizes with 95% confidence intervals.*

The primary analysis will be a repeated measures model. Each patient will contribute four (follow-up) repeated measures to the model, and a compound-symmetry covariance structure will be used to model within-patient correlation. The suitability of this structure was observed in previous work (reference 35 in the protocol). The model will be adjusted for the outcome at baseline and the type of procedure. The other stratification variable, study site, will not be adjusted for since most of the randomised patients were recruited from a single site.

### Secondary Analyses of Primary Outcomes

The primary outcome will be analysed at each of the four follow-up time-points separately. These analyses will be linear regression models, adjusted for the outcome at baseline and the type of procedure.

Two additional models will be fitted with the “highest value” and “lowest value” outcomes as defined in section 4.1.

### Sensitivity and Exploratory Analyses

All analyses will be repeated with the per-protocol set.

### Subgroup Analyses

*Two sensitivity analyses are planned, comparing the primary outcome for … 2. Patients who receive older blood versus younger blood, i.e. those who receive only blood less than 14 days old versus those that receive any blood over 14 days old, on the basis that the proposed intervention is expected to prevent the risks attributed to prolonged blood storage.*

These planned subgroup analyses will not be carried out due to the small numbers of patients who completed the trial.

## Secondary Outcome Analyses

The biomarkers IL-6, MIP-1, MCP-1 and TNF-α will be analysed by the same models as the primary outcome.

The MODS is a long-ordinal variable which will be treated as a continuous variable and analysed in a repeated measures model in the same manner as the primary outcome. In addition, MODS will be analysed according to the “worst-value” method used in [Steiner, 2015]. See section 4.1 for the definition of this derived score.

The EQ-5D summary score will also be treated as a continuous variable and analysed in a similar manner to the primary outcome.

All categorical variables will be analysed using logistic regression adjusted for the type of procedure.

*Time to classification as fit for discharge, ICU and post-operative hospital stay will be analysed as time-to-event data using regression modelling for survival data.*

## Additional Efficacy Analyses

None.

# Safety and Tolerability Analysis

## Drug Exposure

Concomitant medication will be listed and summarised.

## Adverse Events and Tolerability

*Adverse events will be recorded and reported in accordance with the University of Leicester’s and University Hospitals Leicester NHS Trust’s policies for reporting Research Related Adverse Event.*

*In cardiac surgery, post-operative transient complications are not unexpected and are not infrequent. The research team will only notify deaths and ‘unexpected’ non-fatal SAEs to the Trial Sponsor (University of Leicester Research Support Office). Unexpected events are those not listed in the trial protocol or on the case report forms. The sponsor will inform the research team which SAEs should be reported to the REC.*

*The following adverse events are 'expected'* (for details see protocol sect. 8.3)*:*

- Perioperative Ml
- Cardiac arrest
- Haemodynamic support
- Arrhythmias
- Pulmonary complications
- Thromboembolic complications
- Renal complications
- Infective complications
- Gl complications
- Neurological complications
- Bleeding requiring reoperation
- Mediastinitis requiring
- Wound dehiscence requiring rewiring or treatment
- Death

*Data on these adverse events collected during the trial will be regularly reported to the trial DMSC for review.*

*Data on adverse events will be collected from the time of surgery for the duration of the participant's post-operative hospital stay. Data on serious adverse events and follow up data on non-serious adverse drug reactions will be collected from the time of surgery and throughout the 4-6 weeks follow-up period.*

*Frequencies of these adverse outcomes will be tabulated, in line with guidelines for reporting adverse events in trials.*

## Laboratory Data

Any normalising or validating of laboratory values will be carried out before these data are transferred to the CTU.

## Vital Signs, Electrocardiogram and Other Safety Assessments

Vital signs and other safety assessments (e.g. body temperature, pump flow etc.) will be listed individually and summarised by treatment group and in total on each time point.

# References

Bamnote S (2012). Superior gRaphics in Statistical Reports. Phuse. http://www.phusewiki.org/wiki/index.php?title=Superior_gRaphics_in_Statistical_Reports

Steiner M E (2015), Effects of Red-Cell Storage Duration on Patients Undergoing Cardiac Surgery, NEJM, 372:15, 9 April 2015, 1419-1429.

Ranieri M V (2012), Acute Respiratory Distress Syndrome, JAMA, 307(23), June 2012, 2526-2533.

Euroqol http://www.euroqol.org/fileadmin/user_upload/Documenten/PDF/Folders_Flyers/EQ-5D-3L_UserGuide_2015.pdf

# Appendices

## Publication strategy

It is anticipated that the trial results will form part of a wider publication that will include earlier research carried out on the same theme. This earlier work was conducted in collaboration with the Leicester Cardiovascular BRU. The paper is planned to be submitted to the Journal of Clinical Investigation.

## Roles in the analysis

The trial statistician is Tom Morris. Arne Ring was the supervising statistician until 20^th^ February 2015. Cassey Brookes will take over as supervising statistician from June 2015. The CI is Gavin Murphy.

According to CTU SOPs ST-001/002, the trial statistician has the overall responsibility for planning and implementing the trial analysis and its quality assurance. The supervising statistician will contribute to the planning, implementation and validation of the analysis.

According to UoL SOP S-1030, the Chief Investigator must ensure that it is finalised following review by appropriate personnel and approved by the statistician and Sponsor.

Both statisticians and the CI will review and approve the analysis plan.

All SAP authors will be responsible for review and interpretation of the results and will contribute to any publications that are based on these results.

## Technical specification

The technical specifications will be provided as separate documents.

- The Table of contents outlines the titles of the displays for the statistical report (in EXCEL format).
- The SAP appendix document provides display templates and documents details of the programming of the analysis.
- Details of all the outcome measures are provided as a separate document.
